# SARS-CoV-2 vaccination enhances the effector qualities of spike-specific T cells induced by COVID-19

**DOI:** 10.1126/sciimmunol.adh0687

**Published:** 2023-12-08

**Authors:** Curtis Cai, Yu Gao, Sarah Adamo, Olga Rivera-Ballesteros, Lotta Hansson, Anders Österborg, Peter Bergman, Johan K. Sandberg, Hans-Gustaf Ljunggren, Niklas K. Björkström, Kristoffer Strålin, Sian Llewellyn-Lacey, David A. Price, Chuan Qin, Alba Grifoni, Daniela Weiskopf, E. John Wherry, Alessandro Sette, Soo Aleman, Marcus Buggert

**Affiliations:** 1 Department of Medicine Huddinge, Center for Infectious Medicine, Karolinska Institutet, Karolinska University Hospital, Stockholm, Sweden; 2 Department of Hematology, Karolinska University Hospital, Stockholm, Sweden; 3 Department of Oncology-Pathology, Karolinska Institutet, Stockholm, Sweden; 4 Department of Laboratory Medicine, Karolinska Institutet, Stockholm, Sweden; 5 Department of Clinical Immunology and Transfusion Medicine, Karolinska University Hospital, Stockholm, Sweden; 6 Department of Infectious Diseases, Karolinska University Hospital, Stockholm, Sweden; 7 Department of Medicine Huddinge, Infectious Diseases, Karolinska Institutet, Stockholm, Sweden; 8 Division of Infection and Immunity, Cardiff University School of Medicine, University Hospital of Wales, Cardiff, UK; 9 Systems Immunity Research Institute, Cardiff University School of Medicine, University Hospital of Wales, Cardiff, UK; 10 Beijing Key Laboratory for Animal Models of Emerging and Reemerging Infectious Diseases, Institute of Laboratory Animal Science, Chinese Academy of Medical Sciences, Beijing, China; 11 National Health Commission Key Laboratory of Human Disease Comparative Medicine, Comparative Medicine Center, Peking Union Medical College, Beijing, China; 12 Center for Infectious Disease and Vaccine Research, La Jolla Institute for Immunology, La Jolla, California, USA; 13 Department of Medicine, Division of Infectious Diseases and Global Public Health, University of California, San Diego, California, USA; 14 Institute for Immunology, Perelman School of Medicine at the University of Pennsylvania, Pennsylvania, USA; 15 Department of Systems Pharmacology and Translational Therapeutics, Perelman School of Medicine at the University of Pennsylvania, Pennsylvania, USA; 16 Parker Institute for Cancer Immunotherapy, Perelman School of Medicine at the University of Pennsylvania, Pennsylvania, USA

## Abstract

T cells are critical for immune protection against severe COVID-19, but it has remained unclear whether repeated exposure to SARS-CoV-2 antigens delivered in the context of vaccination fuels T cell exhaustion or reshapes T cell functionality. Here, we sampled convalescent donors with a history of mild or severe COVID-19 before and after SARS-CoV-2 vaccination to profile the functional spectrum of hybrid T cell immunity. Using combined single-cell technologies and high-dimensional flow cytometry, we found that the frequencies and functional capabilities of spike-specific CD4^+^ and CD8^+^ T cells in previously infected individuals were enhanced by vaccination, despite concomitant increases in the expression of inhibitory receptors such as PD-1 and TIM3. In contrast, CD4^+^ and CD8^+^ T cells targeting non-spike proteins remained functionally static and waned over time, and only minimal effects were observed in healthy vaccinated donors experiencing breakthrough infections with SARS-CoV-2. Moreover, hybrid immunity was characterized by elevated expression of IFN-γ, which was linked with clonotype specificity in the CD8^+^ T cell lineage. Collectively, these findings identify a molecular hallmark of hybrid immunity and suggest that vaccination after infection is associated with cumulative immunological benefits over time, potentially conferring enhanced protection against subsequent episodes of COVID-19.

## Introduction

Natural infection with severe acute respiratory syndrome coronavirus 2 (SARS-CoV-2) elicits T cell responses against all regions of the viral proteome (1). In contrast, globally adopted vaccination methods focus immune responses on the spike protein alone, primarily aiming to elicit antibodies that neutralize SARS-CoV-2. Accordingly, the combination of infection and vaccination (known as hybrid immunity) elicits T cell responses against both the spike protein and non-spike proteins (2). In retrospective comparisons with previously infected but unvaccinated individuals, hybrid immunity has been associated with lower rates of reinfection (3) and lower rates of hospitalization after reinfection (4), and durable protection against severe disease has largely been maintained despite the emergence of the Omicron variant (5). These observations suggest that hybrid immunity is likely characterized by long-term memory against SARS-CoV-2.

Recurrent antigen exposure in the context of booster vaccination (6) or hybrid immunity (2, 7, 8) has been shown to increase the frequencies of spike-specific T cells in the circulation. Earlier studies nonetheless suggested that repetitive stimulation could lead to T cell dysfunction, especially after severe infection, which has been associated with peripheral lymphopenia (9), higher frequencies of SARS-CoV-2-specific T cells during convalescence (10), and signatures of exhaustion compared with other forms of pneumonia that require hospitalization (11). However, elevated expression of exhaustion markers, such as PD-1, does not necessarily equate with T cell dysfunction (12) and may instead serve as a sign of activation during acute infection. In separate transcriptomic analyses, T cell expression of exhaustion markers was largely equivalent in healthy donors and patients hospitalized with acute COVID-19 (13), and among CD8^+^ T cells targeting SARS-CoV-2, exhaustion signatures were more pronounced in donors with mild versus severe disease (14). Given that mRNA vaccination induces polyfunctional (15) and durable (16) T cell responses more homogenously than infection, and a previous report indicating that CD4^+^ T cells coexpressing interferon (IFN)-γ and interleukin (IL)-10 can arise in the context of hybrid immunity but not after vaccination alone (17), it seems plausible that mRNA vaccination could alter the landscape of established T cell immunity.

Individuals with immunological memory formed during the first pandemic wave of infections (early 2020) and subsequently boosted by vaccination (early 2021) represent a unique case of hybrid immunity generated via recurrent exposure to antigen from the same ancestral Wuhan strain of SARS-CoV-2. We took advantage of this scenario to determine how recurrent stimulation with an identical antigen impacts the frequencies and functional capabilities of CD4^+^ and CD8^+^ T cells targeting the spike protein of SARS-CoV-2. Using *ex vivo* stimulations, high-dimensional flow cytometry, and single-cell RNA-sequencing (scRNA-seq), we identified IFN-γ upregulation as the most consistent and robust signature of hybrid CD4^+^ and CD8^+^ T cell immunity.

## Results

### Vaccination increases spike-specific T cell frequencies after infection with SARS-CoV-2

**Fig. 1:**
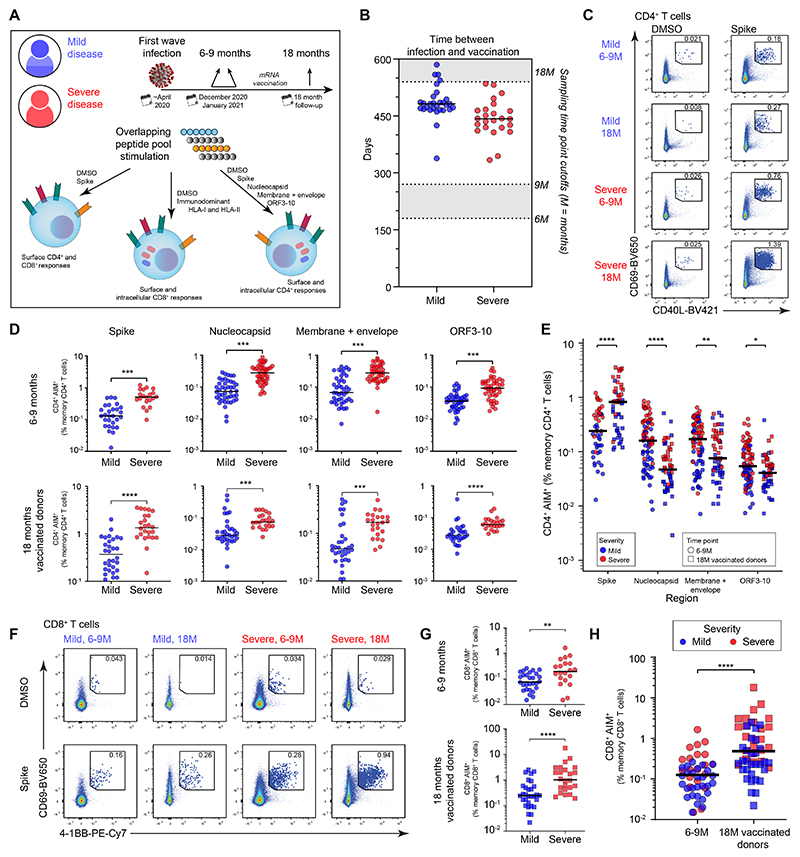
Frequencies of SARS-CoV-2-specific T cells after infection and vaccination. (**A**) Overview of the experimental design. (**B**) Time between vaccination and sampling at 18M. Data are shown for donors with matched samples at 6–9M and 18M. (**C**) Representative flow cytometry plots showing the identification of spike-specific CD4^+^ T cells via activation-induced marker (AIM) expression. (**D**) Frequencies of AIM^+^ memory CD4^+^ T cells targeting different regions of SARS-CoV-2 (spike: intracellular staining, other regions: surface staining). (**E**) Comparison of combined mild and severe AIM^+^ memory CD4^+^ T cell frequencies at 6–9 versus 18M. (**F**) Representative flow cytometry plots showing the identification of spike-specific CD8^+^ T cells via AIM expression. (**G**) Comparison of AIM^+^ memory CD8^+^ T cell frequencies targeting spike between disease severity. (**H**) Comparison of AIM^+^ memory CD8^+^ T cell frequencies targeting spike between time points. Statistical significance was determined by Mann–Whitney U test (D, E, G, H). *P < 0.05, **P < 0.01, ***P < 0.001, ****P < 0.0001. Bars shown median.

To investigate the effects of vaccination after infection with SARS-CoV-2, we first evaluated virus-specific T cell immunity in donors with a confirmed history of mild (non-hospitalized) or severe (hospitalized) COVID-19. Samples of peripheral blood were collected in December 2020 or January 2021, 6–9 months into convalescence (6–9M), and in October 2021, 18 months into convalescence (18M). Matched samples were acquired from 31 donors with a history of mild disease (total *n* = 50) and 24 donors with a history of severe disease (total *n* = 53) (Fig. 1A and table S1). All 6–9M donors were sampled before vaccination was available, and most 18M donors were vaccinated twice between May and August 2021 (mRNA, 52/69; viral vector, 2/69; unknown platform, 5/69; unvaccinated, 10/69) (Fig. 1B and table S1), allowing us to characterize hybrid immunity. Four overlapping peptide pools spanning the spike, nucleocapsid, combined membrane and envelope, and combined open reading frame (ORF) proteins 3–10 were used to assess the functional phenotype of SARS-CoV-2-specific T cells in an activation-induced marker (AIM) assay (Fig. 1A).

At both time points, SARS-CoV-2-specific CD4^+^ T cells identified via coexpression of CD69 and CD40L (Fig. 1C and fig. S1A) were more abundant in donors with severe disease versus mild disease across all tested regions of the viral proteome (Fig. 1D and fig. S1B), consistent with a previous study (10). We additionally confirmed that the contribution from background expression of AIM markers was negligible to the overall frequency of responses (fig. S1, C and D). The effect of vaccination was also evident: CD4^+^ T cell responses directed against the spike protein increased significantly in magnitude after vaccination, whereas CD4^+^ T cell responses directed against other viral proteins remained unchanged or even decreased in magnitude (Fig. 1E). Similar patterns were observed for spike-specific CD8^+^ T cells identified via coexpression of CD69 and 4-1BB (Fig. 1F and fig. S1A). In particular, higher frequencies of spike-specific CD8^+^ T cells were detected in donors with severe versus mild disease (Fig. 1G), and subsequent vaccination increased the frequencies of spike-specific CD8^+^ T cells (Fig. 1H). Notably in the few donors who remained unvaccinated at the 18M time point, both spike-specific CD4^+^ (fig. S2A) and CD8^+^ (fig. S2B) T cell responses remained unchanged from the 6-9M time point and were significantly lower than responses from vaccinated donors. These findings demonstrate that vaccination augments spike-specific CD4^+^ and CD8^+^ T cell frequencies elicited by natural infection with SARS-CoV-2.

## Vaccination reshapes the effector qualities of spike-specific T cells induced by COVID-19

**Fig. 2:**
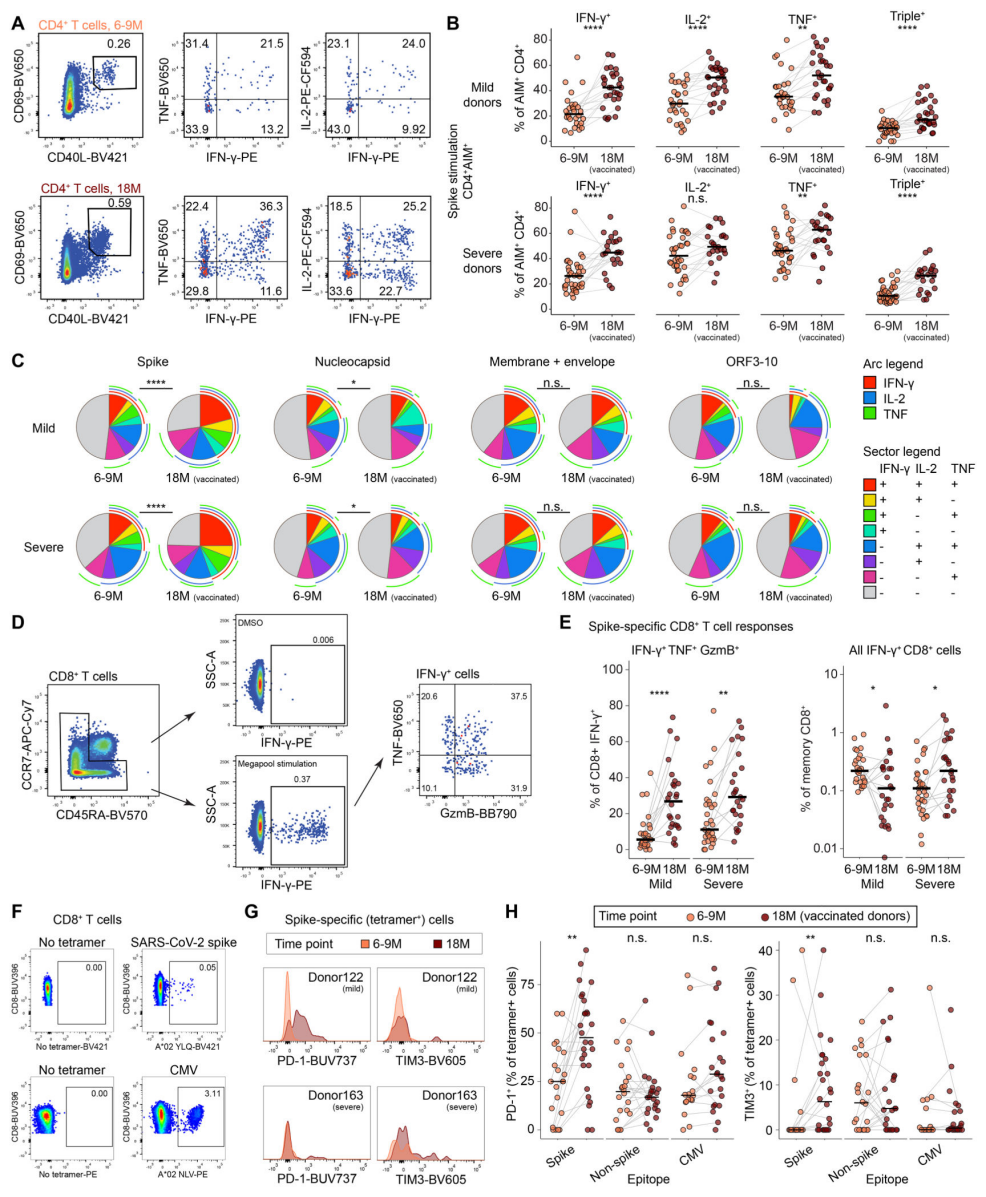
Functionality and inhibitory receptor expression among SARS-CoV-2 spike-specific T cells after infection and vaccination. (**A**) Representative flow cytometry plots showing cytokine expression among AIM^+^ memory CD4^+^ T cells. (**B**) Percentages of AIM^+^ memory CD4^+^ T cells with cytokine expression. (**C**) SPICE analysis of cytokine expression among SARS-CoV-2-specific memory CD4^+^ T cells. (**D**) Representative flow cytometry plots showing cytokine and cytotoxic molecule expression among spike-specific memory CD8^+^ T cells after peptide stimulation. (**E**) Percentages of IFN-γ^+^ spike-specific and total memory CD8^+^ T cells with polyfunctional cytokine and cytotoxic molecule expression after peptide stimulation. (**F**) Representative flow cytometry plots showing the identification of tetramer-binding CD8^+^ T cells specific for SARS-CoV-2 spike or CMV epitopes. Both mild and severe disease donors were pooled and analyzed. (**G**) Representative flow cytometry histograms showing PD-1 or TIM3 expression among spike-specific CD8^+^ T cells. (**H**) Percentages of tetramer-binding CD8^+^ T cells with PD-1 or TIM3 expression. Statistical significance was determined by Mann–Whitney U test (B, E, H) and permutation test (C). n.s. = *P* > 0.05, *P < 0.05, **P < 0.01, ***P < 0.001, ****P < 0.0001. Bars shown median.

To investigate the functionality of spike-specific CD4^+^ T cells after natural infection and subsequent vaccination, we measured the intracellular production of IFN-γ, IL-2, and tumor necrosis factor (TNF) in response to peptide stimulation (Fig. 2A). TNF was the predominant cytokine elicited among spike-specific CD4^+^ T cells at both time points, with slightly lower levels of IL-2 and IFN-γ (Fig. 2B), and the proportion of triple-positive cells increased significantly in vaccinated donors at 18M (Fig. 2B). Spike-specific CD4^+^ T cell polyfunctionality also increased from 6–9M to 18M (Fig. 2C). In contrast, only minor shifts in polyfunctionality were observed among nucleocapsid-specific CD4^+^ T cells, and no significant changes in polyfunctionality were observed among CD4^+^ T cells targeting the combined membrane and envelope proteins or ORF3–10 (Fig. 2C). Of note, robust polyfunctionality was observed before and after vaccination in donors with a history of severe COVID-19, arguing against the occurrence of imprints causing T cell dysfunction long after severe SARS-CoV-2 infection (Fig. 2C and fig. S3A).

CD8^+^ T cell responses were assessed using IFN-γ as a marker of antigen specificity after stimulation with a pool of peptides representing immunodominant epitopes from the spike protein of SARS-CoV-2 (Fig. 2D). The proportions of monofunctional IFN-γ^+^ spike-specific CD8^+^ T cells remained unchanged over time (fig. S3B), whereas the proportions of spike-specific CD8^+^ T cells that coexpressed IFN-γ, TNF, and granzyme B (GzmB) increased at 18M (Fig. 2E). Of note, IFN-γ^+^ spike-specific CD8^+^ T cell frequencies decreased in donors with a history of mild disease but increased in donors with a history of severe disease, indicating a dichotomous effect of vaccination (Fig. 2E).

Recurrent antigen exposure can lead to the upregulation of inhibitory receptors, potentially resulting in T cell exhaustion (18). To investigate this possibility, we used human leukocyte antigen class I (HLA-I) tetramers to first identify SARS-CoV-2-(spike and non-spike) and CMV-(pp65) specific CD8^+^ T cells in the absence of peptide stimulation from both mild and severe disease donors (Fig. 2F and table S2), and next to analyze the surface expression of the exhaustion markers PD-1, TIM3, LAG3, and TIGIT (fig. S3C). The proportions of spike-specific CD8^+^ T cells expressing PD-1 and TIM3 increased after vaccination (Fig. 2, G and H), whereas the proportions of proportions of spike-specific CD8^+^ T cells expressing LAG3 and TIGIT remained unchanged after vaccination (fig. S3D). No changes in inhibitory receptor expression were detected among non-spike-specific or CMV-specific CD8^+^ T cells after vaccination (Fig. 2H and fig. S3D). These results show that vaccination enhances the functional qualities of spike-specific CD4^+^ and CD8^+^ T cells induced by natural infection with SARS-CoV-2, despite the upregulation of inhibitory receptors often associated with exhaustion.

### Single-cell analysis defines the granularity of hybrid spike-specific T cell immunity

**Fig. 3:**
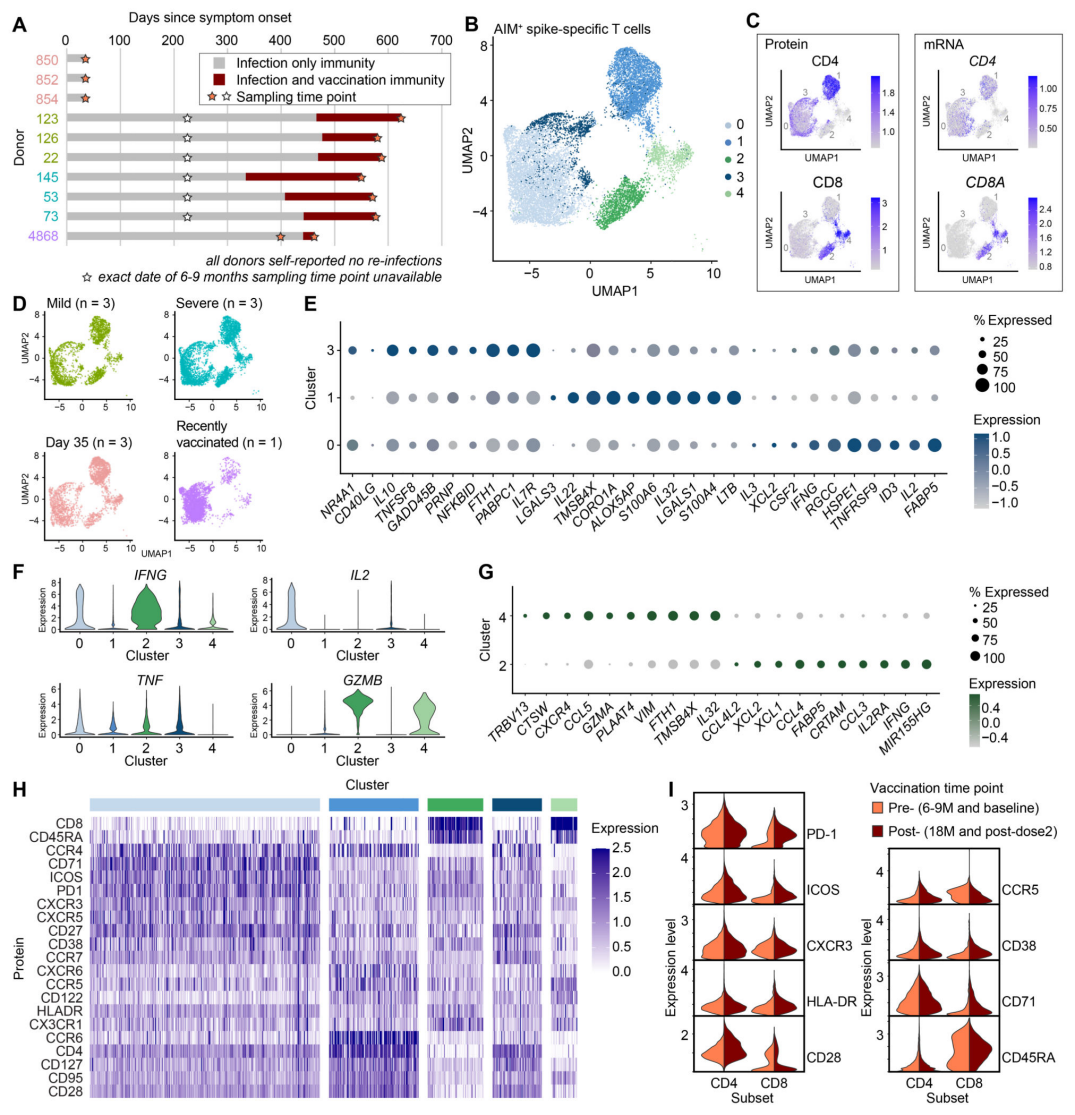
Single-cell analysis of SARS-CoV-2 spike-specific T cells after infection and vaccination. (**A**) Overview of donors and sampling time points selected for scRNA-seq. (**B**) UMAP and clustering of sorted AIM^+^ CD4^+^ and CD8^+^ T cells responding to spike peptide stimulation. (**C**) Expression of CD4 and CD8 at the protein and transcript levels. (**D**) Distribution of conventional AIM^+^ CD4^+^ and CD8^+^ T cells from each donor group. (**E**) Dot plot showing differentially expressed genes in the CD4^+^ T cell clusters. (**F**) Violin plots showing the expression of selected markers previously measured via flow cytometry. (**G**) Dot plot showing differentially expressed genes in the CD8^+^ T cell clusters. (**H**) Heatmap showing protein expression measured via CITE-seq for mild, severe and recently vaccinated donors. (**I**) Violin plots showing the expression of activation markers measured from mild, severe and recently vaccinated donors, separated by time point. The top 10 cluster genes for dot plots were selected by the smallest p-value followed by largest fold-change in the case of ties after Mann–Whitney U test (E and G).

To supplement these findings, we performed scRNA-seq in conjunction with oligo-conjugated antibody staining (CITE-seq) and T cell receptor sequencing (TCR-seq) to profile the global landscape of hybrid immunity. AIM^+^ CD4^+^ and CD8^+^ T cells were sorted via flow cytometry and processed for scRNA-seq from a previously infected donor sampled before and soon after vaccination (*n* = 1), recently infected convalescent donors sampled on day 35 only (*n* = 3), and donors with a history of mild (*n* = 3) or severe (*n* = 3) COVID-19 sampled at 6–9M and 18M (Fig. 3A, fig. S4A, and table S3). We identified five clusters of AIM^+^ conventional CD4^+^ and CD8^+^ T cells after dimensionality reduction via Uniform Manifold Approximation and Projection (UMAP) (Fig. 3, B and C) and the exclusion of NK cells, MAIT cells, γδ T cells, and NKT cells (fig. S4, B to E). Each cluster incorporated cells from each participant group and time point (Fig. 3D and fig. S4F). In general, the frequencies of spike-specific CD4^+^ and CD8^+^ T cells increased after vaccination (fig. S4G), and it should be noted that our sorting strategy was unable to exclude bystander-activated or AIM^+^ expressing cells in the absence of stimulation, although this was likely only consequential for the pre-vaccination CD8^+^ T cell population from donor 126 (where it represented 79% of sorted cells) (fig. S4H).

The largest CD4^+^ T cell cluster (cluster 0) was characterized by effector signatures and abundant expression of the proinflammatory cytokine genes *IFNG* and *IL2* (Fig. 3, E and F). In contrast, clusters 1 and 3 overexpressed the memory-associated genes *LTB* and *IL7R*, respectively (Fig. 3E). *TNF* expression was evenly distributed among clusters, whereas *GZMB* was abundantly expressed in CD8^+^ T cell cluster 2 and, at lower levels, in CD8^+^ T cell cluster 4 (Fig. 3F). Each CD8^+^ T cell cluster was also characterized by a distinct effector signature (cluster 2: *IFNG, CCL3, CCL4*, and *IL2RA*; cluster 4: *GZMA* and *CCL5*) (Fig. 3G).

Using CITE-seq, we quantified various surface markers to link the transcriptional identities of individual clusters with activation, effector, and memory phenotypes defined at the level of protein expression (Fig. 3H and fig. S5A). CD4^+^ T cell cluster 0 (highest CD4^+^ *IFNG* expression) was characterized by relative overexpression of several activation markers, including CD71 and PD-1, whereas CD4^+^ T cell clusters 1 and 3 exhibited Th17-like memory (CCR6^+^CD127^+^) and memory (CD127^+^) phenotypes, respectively. Analogously, CD8^+^ T cell cluster 2 (highest CD8^+^ *IFNG* expression) also exhibited relative overexpression of several activation markers, including PD-1, ICOS, HLA-DR, and CD71. Vaccination further increased the expression of PD-1 (median at pre: 0.33, median at post: 0.93, Mann Whitney U test p-value: 2.18×10^-34^) in the CD8^+^ T cell lineage (Fig. 3I and fig. S5B), confirming our previous observations using HLA-I tetramers in conjunction with conventional flow cytometry (Fig. 2, G and H). These results demonstrate concordant phenotypic heterogeneity at the levels of gene and protein expression and reveal the molecular landscape of spike-specific CD4^+^ and CD8^+^ T cells in the context of hybrid immunity against SARS-CoV-2.

### *IFNG* upregulation is a marker of hybrid spike-specific CD4^+^ T cell immunity

**Fig. 4:**
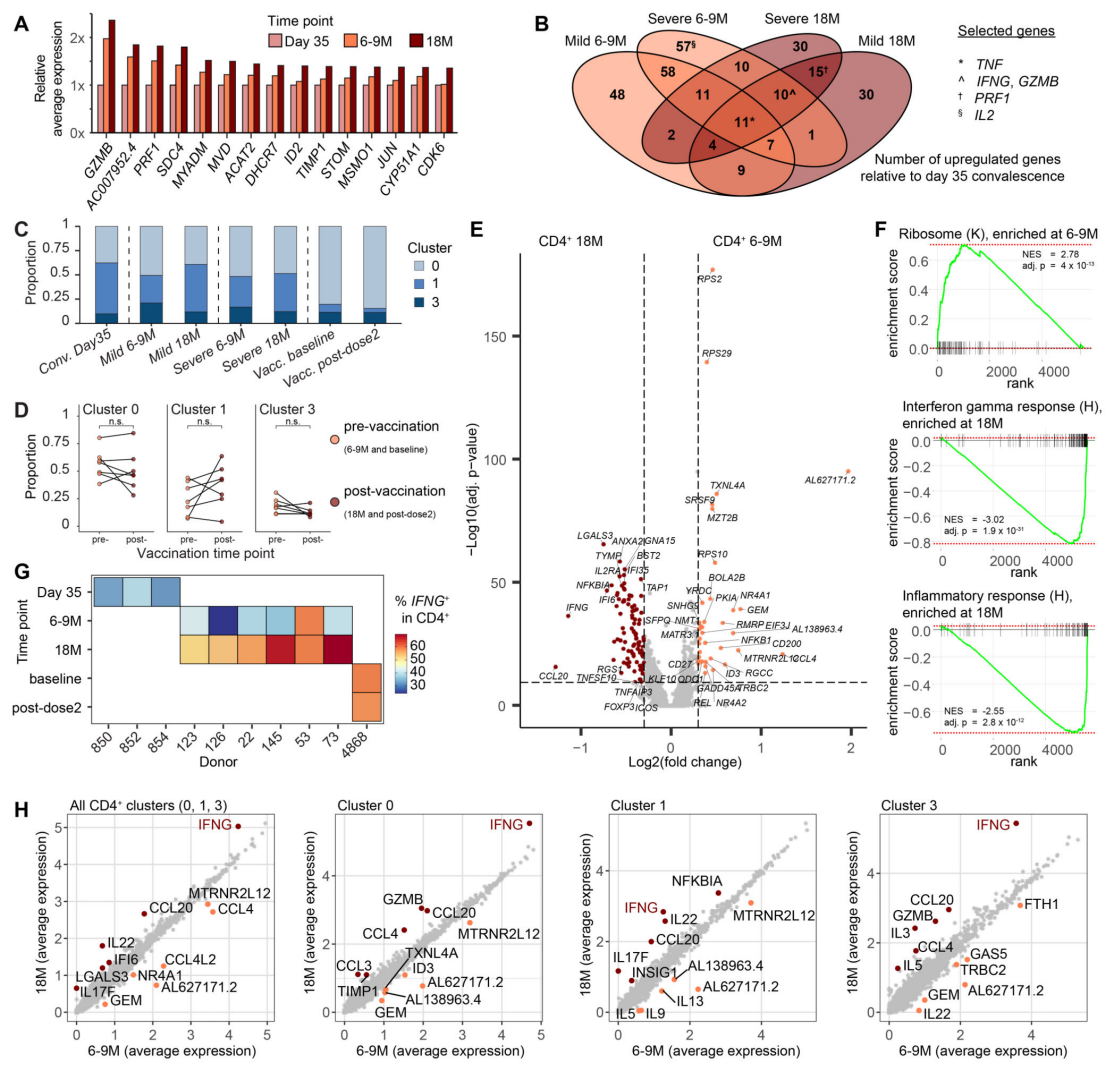
SARS-CoV-2 spike-specific CD4^+^ T cells exhibit a proinflammatory profile in the setting of hybrid immunity. (**A**) Ranked comparison of average transcription expression in AIM^+^ CD4^+^ T cells grouped by time point. (**B**) Venn diagram showing differentially expressed genes shared among groups compared with AIM^+^ CD4^+^ T cells from recently infected donors sampled on day 35. (**C**) Proportions of AIM^+^ CD4^+^ T cells belonging to each CD4^+^ T cell cluster. (**D**) Comparison of the proportion of cells belonging to each CD4^+^ T cell cluster from mild, severe, and recently vaccinated donors. (**E**) Volcano plot showing differentially expressed genes between AIM^+^ CD4^+^ T cells at 6–9M versus 18M. Dashed lines indicated adjusted p-value = 0.05 and fold change = ±0.25. (**F**) Gene set enrichment analysis of differentially expressed genes between AIM^+^ CD4^+^ T cells at 6–9M versus 18M showing significant hits from the KEGG (K) and Hallmark (H) pathways. NES: normalized enrichment score. (**G**) Percentages of AIM^+^ CD4^+^ T cells with expression of *IFNG*. (**H**) Comparison of average gene expression for all AIM^+^ CD4^+^ T cells versus individual CD4^+^ T cell clusters separated by time point. Labels identify the top six genes with the largest differences in expression. Statistical significance was determined by paired Wilcoxon Signed-Rank test (D), Mann–Whitney U test (E) and Broad GSEA test (F). Adjusted p-values calculated using the Bonferroni (E) and Benjamini–Hochberg (F) methods. n.s. = *P* > 0.05.

Using this combined dataset as a basis for exploration, we hypothesized that stepwise changes in gene expression from day 35 to 6–9M and from 6–9M to 18M might reflect the sequential effects of long-term memory formation and vaccination, respectively. Averaging transcript abundance across each time point and ordering gene expression by fold change revealed that cytotoxic molecules, including *GZMB* and *PRF1*, were progressively upregulated in the CD4^+^ T cell lineage (Fig. 4A). To determine if these differences held across the spectrum of disease severity, we next performed a differential gene expression analysis of spike-specific CD4^+^ T cells sampled on day 35 and at 6–9M and 18M after mild or severe COVID-19. Eleven genes were upregulated in all groups, including *TNF*, and other effector molecules, including *IFNG, GZMB*, and *PRF1*, were upregulated after vaccination in spike-specific CD4^+^ T cells obtained from donors with a history of mild or severe COVID-19 (Fig. 4B).

In each donor group, the proportions of cells belonging to CD4^+^ clusters 0, 1, and 3 remained stable over time (Fig. 4, C and D). Samples from the convalescent day 35 donors typically had the largest proportion of cluster 1 cells and the recently vaccinated donor had the largest proportion of cluster 0 cells and this distribution was similarly maintained after vaccination. A single Donor 22 exhibited a substantial expansion of spike-specific CD4^+^ T cells in cluster 1 after vaccination (fig. S6A). We next performed differential gene expression analysis and identified *CCL20, IFNG*, and *LGALS3* as the most highly upregulated transcripts at 18M (Fig. 4E). In addition, gene set enrichment analysis (GSEA) showed overrepresentation of the Kyoto Encyclopedia of Genes and Genomes (KEGG) ribosome pathway at 6–9M (Fig. 4F), which may be associated with an early differentiated phenotype poised for activation and effector functionality (19). GSEA also confirmed enrichment of *IFNG* and inflammatory response gene sets at 18M (Fig. 4F and fig. S6B). Indeed, the proportions of cells expressing *IFNG* were elevated after vaccination in all donors with a history of mild or severe disease (Fig. 4G and fig. S6C), but in contrast to a previous report (17), we found no evidence of contemporaneous increases in the frequencies of CD4^+^ T cells expressing IL-10 (fig. S6, D and E). Average gene expression calculations for every transcript segregated by time point further revealed that *IFNG* expression was consistently elevated at 18M, irrespective of whether clusters 0, 1, and 3 were considered together or separately (Fig. 4H). These results indicate that spike-specific CD4^+^ T cells shift toward a proinflammatory *IFNG*^+^ phenotype after vaccination against COVID-19.

### Extensive transcriptome redistribution defines hybrid spike-specific CD8^+^ T cell immunity

**Fig. 5:**
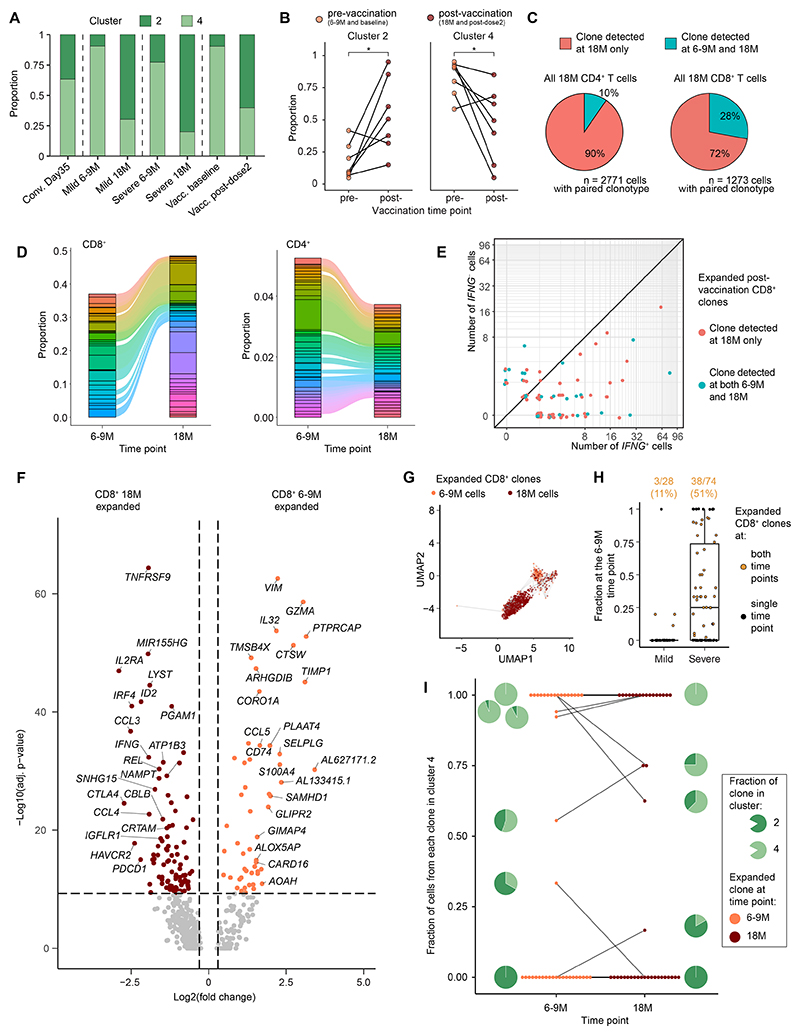
SARS-CoV-2 spike-specific CD8^+^ T cells shift to a proinflammatory phenotype via clonal recruitment or expansion after vaccination in the setting of hybrid immunity. (**A**) Proportions of AIM^+^ CD8^+^ T cells belonging to each CD8^+^ T cell cluster. (**B**) Comparison of the proportion of cells belonging to each CD8^+^ T cell cluster from mild, severe, and recently vaccinated donors. (**C**) Proportions of AIM^+^ CD8^+^ T cells with paired TCRα and TCRβ chain sequences detected only at 18M or at 6–9M and at 18M. (**D**) Alluvial plots showing shared AIM^+^ CD8^+^ T cell clonotypes before and after vaccination. (**E**) *IFNG* expression among expanded AIM^+^ CD8^+^ T cell clonotypes after vaccination. (**F**) Volcano plot showing differentially expressed genes between clonally expanded AIM^+^ CD8^+^ T cells at 6–9M versus 18M. Dashed lines indicated adjusted p-value = 0.05 and fold change = ±0.25. (**G**) UMAP plot of clonally expanded AIM^+^ CD8^+^ T cells. Lines connect shared clonotypes. (**H**) Comparison of expanded AIM^+^ CD8^+^ T cell clonotypes from all time points showing fractional representation at 6–9M. The percentages of AIM^+^ CD8^+^ T cell clonotypes detected at both time points are shown above. (**I**) Fractional representation of AIM^+^ CD8^+^ T cell clonotypes present at both time points in cluster 4. Lines connect identical clonotypes. Statistical significance was determined by paired Wilcoxon Signed-Rank test (B) and Mann–Whitney U test (and F). Adjusted p-values calculated using the Bonferroni method (F). *P < 0.05.

We next compared the transcriptomes of spike-specific CD8^+^ T cells (composed of clusters 2 and 4) before and after vaccination. SARS-CoV-2 booster vaccination increases the frequencies of spike-specific CD8^+^ T cells to a substantially greater extent than the frequencies of spike-specific CD4^+^ T cells, indicating greater responsiveness to recurrent antigen exposure (20). We noted that in addition to these previous observations, CD8^+^ T cells were significantly redistributed from cluster 4 to cluster 2 when comparing pre- and post-vaccination time points irrespective of initial disease severity (Fig. 5, A and B and fig. S6F). Notably, Donor 854 from the cohort of convalescent day 35 donors already had a majority population of cluster 2 cells in the absence of vaccination (fig. S6F). Cluster 2, which contributed the majority of cells after vaccination, was characterized by greater expression of classical type 1 proinflammatory (*IFNG* and *TNF*), chemotactic (*CCL3* and *CCL4*), and inflammatory (*XCL1* and *XCL2*) chemokines/cytokines (Fig. 3G).

Due to the large transcriptional shift among CD8^+^ T cells, we attempted to identify the origins of spike-specific CD8^+^ T cell clonotypes after vaccination using an integrated approach combining transcriptomics and TCR-seq. Nucleotide identity across rearranged TCRα and TCRβ sequences in the Complementarity-determining region 3 (CDR3) was used to define clonality. Spike-specific CD8^+^ T cell clonotypes were more commonly shared between the two time points compared with CD4^+^ T cell clonotypes (Fig. 5C and fig. S7A) and more commonly expanded after vaccination (Fig. 5D). Most spike-specific CD8^+^ T cell clonotypes also expressed *IFNG* at 18M (Fig. 5E). However, we found no evidence to suggest that either clonotypes recruited by the original infection or clonotypes recruited in response to subsequent vaccination preferentially expressed *IFNG* (Fig. 5E and fig. S7B), instead observing that expanded clonotypes were enriched for effector molecules (*IFNG, TNF, CCL3*, and *CCL4*) at 18M (Fig. 5F).

To further understand this dichotomy, we compared the transcriptomes of spike-specific CD8^+^ T cell clonotypes that were present at one time point with the transcriptomes of spike-specific CD8^+^ T cell clonotypes that were present at both time points, performing differential gene expression analysis and GSEA (fig. S8, A to C). In contrast to clonotypes that were present at both time points, which overexpressed effector genes (*XCL1, XCL2*, and *NKG7*), clonotypes present only at 18M overexpressed the memory-associated marker *LTB* (fig. S8A). These differences were corroborated by the enrichment of cytotoxicity and inflammatory pathways among clonotypes detected at both time points and the enrichment of IFN signaling responses among clonotypes detected only at 18M (fig. S8, B and C).

The extensive transcriptomic shift from cluster 4 to 2 raised the possibility that vaccination promoted the differentiation of spike-specific CD8^+^ T cells initially detected at 6–9M. To test this hypothesis, we analyzed expanded clonotypes detected at 6–9M and 18M, linking cells that shared identical TCRs (Fig. 5G). Approximately half of all clonotypes from donors with a history of severe disease were present at both time points compared with only 11% of all clonotypes from donors with a history of mild disease (Fig. 5H). Interestingly, vaccination did not appear to cause a direct migration from cluster 4 (*IFNG*^−^) to cluster 2 (*IFNG*^+^), as only 3/41 clonotypes present at 6–9M were more commonly represented in cluster 2 at 18M (Fig. 5I). Together, these data suggest that vaccination increases the proportions spike-specific CD8^+^ T cells that express *IFNG*, likely via the expansion of existing *IFNG*^+^ cells and/or the *de novo* recruitment of *IFNG*^+^ cells rather than via functional remodeling of *IFNG*^−^ cells into *IFNG*^+^ cells after induction.

### Infection and vaccination timelines affect the quality and quantity of spike-specific T cells

To extend these findings, we investigated how booster vaccinations impacted spike-specific CD4^+^ and CD8^+^ T cells in donors with or without a prior history of infection with SARS-CoV-2. Initially, we studied a cohort of patients with chronic lymphocytic leukemia (CLL) undergoing treatment with ibrutinib, which suppresses B cell proliferation and survival by irreversibly inhibiting Bruton’s tyrosine kinase (BTK), all of whom (*n* = 7) had received two booster vaccinations (totaling four vaccine doses) after infection with SARS-CoV-2 (fig. S9A and table S4). The frequencies of spike-specific CD8^+^ T cells increased significantly after booster vaccination compared with baseline (fig. S9B). In contrast, the frequencies of spike-specific CD4^+^ T cells remained largely unchanged after vaccination (fig. S9C) and approximated those observed in non-CLL donors with a history of mild or severe disease before vaccination (0.29–1.33%) (Fig. 1D), potentially reflecting a ceiling effect (6). These cells nonetheless showed no signs of functional exhaustion and more commonly expressed IFN-γ after vaccination (fig. S9D). Of note, the frequencies of total memory CD8^+^ T cells expressing IFN-γ was higher in 6/7 donors after vaccination, although this trend did not reach statistical significance (fig. S9E).

**Fig. 6:**
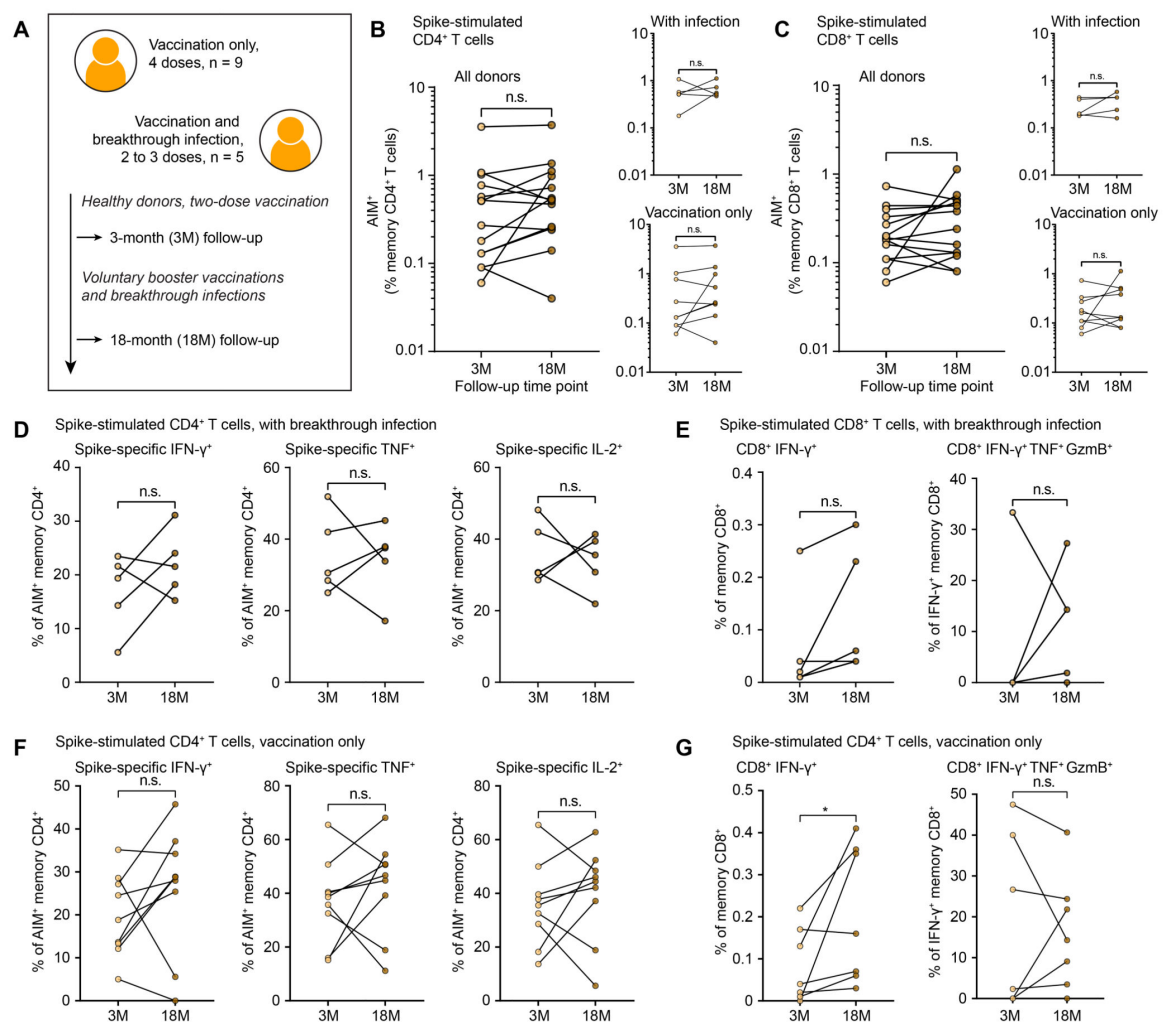
SARS-CoV-2 spike-specific T cells are minimally impacted by breakthrough infection or booster vaccination. (**A**) Overview of healthy vaccinated donors and sampling time points. (**B**) Frequencies of AIM^+^ memory CD4^+^ T cells in healthy vaccinated donors. (**C**) Frequencies of AIM^+^ memory CD8^+^ T cells in healthy vaccinated donors. (**D**) Percentages of AIM^+^ memory CD4^+^ T cells expressing cytokines after breakthrough infection. (**E**) Percentages of total memory and IFN-γ^+^ memory CD8^+^ T cells expressing cytokines and cytotoxic molecules after breakthrough infection. (**F**) Percentages of AIM^+^ memory CD4^+^ T cells expressing cytokines after vaccination. (**G**) Percentages of AIM^+^ memory CD8^+^ T cells expressing cytokines and cytotoxic molecules after vaccination. Statistical significance was determined by paired Wilcoxon Signed-Rank Test (B, C, D, E, F, G). n.s. = *P* > 0.05, *P < 0.05.

We then evaluated spike-specific CD4^+^ and CD8^+^ T cell responses in healthy donors (*n* = 14) enrolled in a vaccination cohort (21), sampling at 3M (non-infected) and 18M after the second dose (Fig. 6A and table S5). At the later time point, 9/14 donors had received 3–4 doses of an mRNA vaccine without contracting SARS-CoV-2, whereas 5/14 donors had received 2–3 doses of an mRNA vaccine and experienced a breakthrough infection with SARS-CoV-2. We found no significant differences in the frequencies of spike-specific CD4^+^ or CD8^+^ T cells at 3M versus 18M (Fig. 6, B and C) or the proportions of cytokine-producing CD4^+^ or CD8^+^ T cells associated with breakthrough infection (Fig. 6, D and E) or vaccination alone (Fig. 6, F and G). Increased frequencies of IFN-γ^+^ CD8^+^ T cells were nonetheless observed in the contexts of breakthrough infection for all donors (Fig. 6E), albeit without achieving statistical significance, and vaccination alone (Fig. 6G). These findings show that booster vaccination in the absence of prior infection does not significantly enhance the frequencies and overall functional qualities of spike-specific CD4^+^ and CD8^+^ T cells in the circulation (fig. S10).

## Discussion

SARS-CoV-2 vaccination has saved tens of millions of lives during the current pandemic by reducing the incidence of severe COVID-19 (22). In particular, durable protection from severe disease has been observed in patients with hybrid immunity (5), indicating that long-term immunological memory could play a key role. In this study, we assessed the frequencies and functional qualities of SARS-CoV-2-specific CD4^+^ and CD8^+^ T cells in donors with a history of mild or severe disease before and after mRNA vaccination. We found that non-spike-specific CD4^+^ T cells retained their original functional characteristics but declined numerically over time. In contrast, the frequencies and functional capabilities of spike-specific CD4^+^ and CD8^+^ T cells increased after vaccination, despite the upregulation of inhibitory receptors among spike-specific CD8^+^ T cells compared with baseline. Booster vaccination also enhanced the functional profiles of spike-specific CD4^+^ T cells and the frequencies of spike-specific CD8^+^ T cells in previously infected patients with CLL. However, these changes were not consistently observed in previously vaccinated but uninfected individuals after boosting or breakthrough infection, suggesting that recurrent antigen exposure in this context may simply counteract waning immunity (23, 24) and not be an indication of which order of antigen exposure may induce optimal immunity. These collective findings demonstrate that vaccination can enhance T cell immunity without necessarily inducing T cell exhaustion after infection with SARS-CoV-2.

T cells often upregulate multiple inhibitory receptors in response to severe COVID-19 (25). This phenomenon has been associated with functional exhaustion in the context of chronic viral infections, such as HIV-1 (26), and likely reflects an immunological adaptation to ongoing antigen exposure (27). However, inhibitory receptor expression during early infection with SARS-CoV-2 may simply demarcate *de novo* virus-specific T cell activation (28), especially given the high levels of viral replication that accompany severe disease (29). Our data align with this notion. We also found that spike-specific and nucleocapsid-specific CD4^+^ T cells were more polyfunctional after severe versus mild COVID-19. Moreover, vaccination enhanced the functional profiles of spike-specific CD4^+^ and CD8^+^ T cells in donors with a history of severe disease and further increased inhibitory receptor expression among spike-specific CD8^+^ T cells, consistent with robust immunological memory rather than exhaustion. These observations suggest that vaccination may help protect convalescent individuals recovering from severe disease against future encounters with SARS-CoV-2.

SARS-CoV-2 vaccination has been associated with dysfunctional T cell immunity (30, 31). To assess this possibility in more detail, we used *ex vivo* peptide stimulation in conjunction with flow cytometry and scRNA-seq to profile SARS-CoV-2-specific CD4^+^ and CD8^+^ T cells functionally, phenotypically, and transcriptionally. We found that spike-specific CD4^+^ and CD8^+^ T cells overexpressed many proinflammatory chemokines and cytokines after vaccination, including IFN-γ. These data align with previous work showing that mRNA vaccination induces strong inflammatory responses (32) and potentially drives spike-specific CD4^+^ T cell clonotypes into a T_H_1-like differentiation program (33). However, we did not identify spike-specific CD4^+^ T cells coexpressing IFN-γ and IL-10, which could potentially balance inflammatory signals in the context of hybrid immunity (17). Of note, the scRNA-seq data indicated distinct transcriptional remodeling of the CD4^+^ and CD8^+^ T cell lineages after vaccination. In particular, the overall memory response was altered among spike-specific CD4^+^ T cells and skewed toward greater cytokine production (*e.g*., *IFNG* and *TNF*), without necessarily affecting T_H_1-like chemokine receptor expression patterns (*e.g*., *CXCR3*) or cluster identity. In contrast, spike-specific CD8^+^ T cells underwent a marked transcriptional shift toward an *IFNG*^+^ response profile, feasibly enabling anamnestic responses characterized by the rapid production of IFN-γ. We also found that the *IFNG* phenotype among spike-specific CD8^+^ T cells was tightly linked with the expression of distinct TCRs. Accordingly, vaccination likely expands the relative frequencies of existing *IFNG*^+^ clonotypes and/or preferentially recruits new *IFNG*^+^ clonotypes, although we were unable to distinguish between these possibilities by definitively identifying the origins of emerging *IFNG*^+^ clonotypes at 18M.

T cell responses that simultaneously deliver multiple antiviral effector functions have been associated with clearance or enhanced immune control of many viral infections, but coexpression of IL-10 can limit immunopathology (34, 35) and has been associated with asymptomatic COVID-19 (36). We found that IFN-γ expression induced by mRNA vaccination was a hallmark of hybrid immunity. IFN-γ exhibits potent antiviral effects and has been used successfully to treat immunocompromised patients infected with SARS-CoV-2 (37). In addition, low serum levels of IFN-γ, together with advanced age and a lack of vaccination, have been associated independently with the risk of contracting severe COVID-19 (38). CD8^+^ T cells are major producers of IFN-γ, which suppresses viral replication via the upregulation of interferon-stimulated genes (ISGs) that subsequently enhance antigen presentation and recruit multiple immune cell types to the site of infection (39). Moreover, recent data indicate that mRNA vaccination induces protection in B cell-deficient mice, attributable to T cell immunity via the production of IFN-γ (40). Enhanced T cell effector functionality with a balanced inflammatory profile in the setting of hybrid immunity could therefore mediate durable protection against severe COVID-19.

There are several limitations to our study. First, all donors with a history of mild or severe disease were infected with and vaccinated against the Wuhan strain of SARS-CoV-2, which allowed us to track immune responses specific for a defined antigen over time but nonetheless excluded similar analyses of individuals infected with more recent variants and/or vaccinated with booster formulations against subvariants of Omicron. However, this is likely a minor consideration, given that ancestral T cells efficiently cross-recognize the Omicron variant of SARS-CoV-2 (41–43). Second, we did not actively match individuals with a history mild or severe disease for comorbidities, potentially confounding associations between the nature of the immune response and the initial severity of COVID-19. Third, our recently vaccinated donor harbored a small population of spike-specific CD8^+^ T cells before and after vaccination, precluding the possibility of tracking individual clonotypes to discriminate between anamnestic and *de novo* clonal expansions over time. Fourth, our scRNA-seq experiments were potentially confounded by gender bias, given that all three donors with a history of mild disease were male, whereas just one donor with a history of severe disease was male. Fifth, limited cell numbers were available for scRNA-seq, limiting our ability to discern the origins of newly detected *IFNG*^+^ spike-specific CD8^+^ T cell clonotypes after infection or subsequent vaccination. Finally, the limited number of statistically significant differences observed in the CLL and vaccination cohorts may have reflected the smaller sample sizes tested.

In summary, we have shown that spike-specific but not non-spike specific CD4^+^ and CD8^+^ T cells become more polyfunctional in previously infected individuals after mRNA vaccination, irrespective of inhibitory receptor expression and the initial severity of COVID-19. We have also demonstrated that upregulated expression of IFN-γ among spike-specific CD4^+^ and CD8^+^ T cell clonotypes is a common hallmark of vaccine-induced hybrid immunity. Collectively, these data indicate that vaccination after infection is associated with cumulative immunological benefits over time, potentially conferring enhanced protection against subsequent exposure to SARS-CoV-2.

## Materials and Methods

### Study design

SARS-CoV-2-specific T cell responses in convalescent donors were detected using overlapping peptide pools spanning the spike protein and other viral regions to induce the upregulation of activation-induced markers. T cell responses were characterized using flow cytometry and scRNA-seq after natural infection and after subsequent mRNA vaccination to measure phenotypic changes at the protein and transcriptional levels after repetitive antigen exposure. Equivalent analyses were performed in patients with altered B cell functionality and healthy donors with breakthrough infections to calibrate the data as a function of multiple vaccinations or the order of antigen exposure.

### Patient samples

Venous blood samples were obtained from convalescent donors after infection with SARS-CoV-2, confirmed via RT-PCR testing at the Karolinska University Hospital, Stockholm, Sweden. Disease severity was stratified as mild or severe based on hospitalization for COVID-19 (table S1). All donors were infected during the first wave of SARS-CoV-2, peaking in March and April 2020. Samples were collected 6–9 months after COVID-19 (6–9M) and/or 18 months after COVID-19 (18M). Participants were vaccinated primarily with mRNA formulations offered by The Public Health Agency of Sweden. Additional venous blood samples were obtained from convalescent donors on day 35 after symptom onset (*n* = 3) and from patients undergoing treatment with ibrutinib for CLL (*n* = 7). The latter were sampled after natural infection and after a fourth vaccine dose (table S4). Six of these donors were hospitalized with COVID-19. A further donor with previously confirmed infection was sampled 2 weeks before and 2 weeks after double vaccination. Healthy vaccinated controls (*n* = 14) were recruited via the COVAXID Study (21) as detailed in table S5. PBMCs were isolated via standard density gradient centrifugation and cryopreserved in fetal bovine serum (FBS) containing 10% dimethyl sulfoxide (DMSO). Written informed consent was obtained from all donors in accordance with the principles of the Declaration of Helsinki. The study was approved by the Swedish Ethical Review Authority and by regional ethics boards at the University of California, San Diego, USA.

### Peptides

Surface markers were analyzed after stimulation with peptide pools (15mers overlapping by 11 amino acids) spanning the entire spike protein of SARS-CoV-2 (Peptides&Elephants GmbH). Functional analyses of CD4^+^ T cells via the identification of intracellular markers were performed after stimulation with peptide pools (20mers overlapping by 10 amino acids) spanning the entire spike, nucleocapsid, combined membrane and envelope, and combined ORF3–10 proteins of SARS-CoV-2 (Sigma-Aldrich). AIM expression among unstimulated CD8^+^ T cells was prohibitively high after staining intracellularly. Functional analyses of CD8^+^ T cells via the identification of intracellular markers were therefore performed using a pool of HLA-I-restricted and HLA-II-restricted peptides representing immunodominant epitopes from the spike protein of SARS-CoV-2 (Sigma-Aldrich) and limited to the detection of IFN-γ. All peptide sequences were based on the ancestral Wuhan strain of SARS-CoV-2. Lyophilized peptides were reconstituted at a stock concentration of 10 mg/ml in DMSO and diluted to 100 μg/ml in phosphate-buffered saline (PBS).

### Activation-induced marker assay

PBMCs were thawed quickly, resuspended in complete medium in the presence of DNase I (10 U/ml, Sigma-Aldrich), and rested at 1 × 10^6^ cells/well in 96-well U-bottom plates (Corning) for 3 h at 37°C. For surface analyses, the medium was supplemented with anti-CXCR5–BB515 (clone RF8B2, BD Biosciences) and unconjugated anti-CD40 (clone HB14, Miltenyi Biotec), followed 15 min later by the relevant peptides (each at 0.5 μg/ml). Cells were then incubated for 12 h at 37°C. For intracellular analyses, the medium was supplemented with anti-CXCR5–BB515 (clone RF8B2, BD Biosciences), followed 15 min later by the relevant peptides (each at 0.5 μg/ml) and a further 1 h later by brefeldin A (1 μg/ml, Sigma-Aldrich) and monensin (0.7 μg/ml, BD Biosciences). Cells were then incubated for 9 h at 37°C. Negative control wells lacked peptides and contained volume-equivalent DMSO.

### Flow cytometry

Cells were stimulated as described above, washed in FACS buffer (PBS supplemented with 2% FBS and 2 mM EDTA), and stained as detailed in tables S6 and S7. Stained cells were then fixed with 1% paraformaldehyde in PBS and acquired using a FACSymphony A5 (BD Biosciences). Data were analyzed using FlowJo version 10 (FlowJo LLC). Healthy vaccinated controls were evaluated using a reduced surface panel, excluding CCR4, CCR6, and CXCR3 from table S6, and a reduced surface and intracellular panel, excluding CCR4, CCR6, CXCR3, CD38, and PD-1 from table S7. Stimulation indices were calculated by dividing the frequencies of AIM^+^ cells in experimental wells containing the relevant peptides by the corresponding frequencies of AIM^+^ cells in negative control wells containing volume-equivalent DMSO. Analyses of intracellular AIM^+^ CD4^+^ T cells or IFN-γ^+^ CD8^+^ T cells were limited to populations with a minimum of 10 events in the corresponding target gate. Functional profiles were compared using a permutation test and visualized in SPICE version 6 (https://niaid.github.io/spice/).

### Tetramers

HLA-I tetramers were generated as described previously (44). The following specificities were used in this study: SARS-CoV-2 spike A*0201 YLQPRTFLL, SARS-CoV-2 spike A*2402 QYIKWPWYI, SARS-CoV-2 spike B*0702 SPRRARSVA, SARS-CoV-2 nucleocapsid A*0201 LLLDRLNQL, SARS-CoV-2 nucleocapsid B*0702 SPRWYFYYL, SARS-CoV-2 ORF3a A*0201 LLYDANYFL, SARS-CoV-2 ORF3a A*0201 ALSKGVHFV, CMV pp65 A*0201 NLVPMVATV, and CMV pp65 B*0702 TPRVTGGGAM. Donors were typed via flow cytometry using anti-HLA-A2–PE-Cy7 (clone BB7.2, BioLegend), anti-HLA-A24–FITC (clone 220, MBL International), and anti-HLA-B7–APC (clone BB7.1 BioLegend). The relevant tetramers were then used in conjunction with a panel of surface markers to identify and phenotype virus-specific CD8^+^ T cells as detailed in table S8.

### Single-cell RNA sequencing

PBMCs were stimulated with spike peptides as described above and stained as detailed in table S9. Stained cells were then sorted as lymphocytes/singlets/viable/CD4^+^/CD69^+^CD40L^+^ or lymphocytes/singlets/viable/CD8^+^/CD69^+^4-1BB^+^ populations using an MA900 Multi-Application Cell Sorter (Sony Biotechnology). Cells stained with unique hashing antibodies were sorted from up to three samples into a single microfuge tube (Sarstedt). Pooled samples were loaded onto a Chromium Single Cell Chip (10x Genomics). Libraries were prepared using a Chromium Next GEM Single Cell V(D)J Reagent Kit v1.1 (10x Genomics). Sequencing was performed using an 8-base index read, a 26-base read 1 containing barcodes and unique molecular identifiers (UMIs), and a 98-base read 2 containing transcript sequences to a depth of approximately 50,000 to 90,000 reads per cellular barcode on a NovaSeq6000 SP100 Flow Cell (Illumina).

### Single-cell RNA analysis

Sequencing outputs were delivered as demultiplexed fastq files and processed into expression matrices using the multi command in CellRanger version 6.1.1 (10x Genomics). Expression data (gene, protein, and hashtag) were imported using the Read10X function in Seurat version 4.1.1 (45). Cell inclusion required fewer than 6% of reads aligned to mitochondrial genes and a distinct gene expression range from 1000 to 5700. Transcript expression was normalized using the LogNormalize option from the NormalizeData function in Seurat version 4.1.1. Antibody and hashtag data were transformed using the centered log-ratio approach. Hashtag oligo (HTO) demultiplexing was performed using the HTODemux function in Seurat version 4.1.1. Antibody detection was used to group cells according to the expression of CD4 or CD8. TCR data were imported using the import_vdj command in the djvdj package in R and filtered to retain only the most commonly expressed α and β chain sequences, grouping cells as a single clonotype if these sequences matched exactly. Alluvial plots for shared clonotypes between time points were generated for the top 50 sequences using the compareClonotypes function in scRepertoire version 1.4.0 (46). Data from additional samples processed in distinct batches were integrated using the SelectIntegrationFeatures, FindIntegrationAnchors, and IntegrateData functions in Seurat version 4.1.1. Cells were clustered using the FindNeighbors function with the first 20 principal component dimensions and a resolution of 0.2 for the FindClusters function in Seurat version 4.1.1. Dimensionality reduction was performed using UMAP. Irrelevant cells expressing CD8 were eliminated from the analysis by removing NK cells (UMAP cluster 5), MAIT cells (UMAP cluster 6), γδ T cells (expressing *TRGV3, TRGV9, TRDV1*, or *TRDV3*), and NKT cells (coexpressing *TRAV10* and *TRAJ18*). Differential gene expression analyses were performed using the FindMarkers and FindAllMarkers functions with the Wilcoxon rank-sum test and bonferroni correction in Seurat version 4.1.1. The top ten most significant genes ordered by adjusted p-value after differential gene expression were selected for visualization on dot plots. GSEA was performed using the fgsea package in R with 5000 permutations and gene sets downloaded from the MSigDB. Average gene expression was calculated using the AverageExpression function in Seurat version 4.1.1. Figures were prepared using ggplot2 and Seurat version 4.1.1 in R.

### Statistics

Statistical analyses were performed using Prism software version 9 (GraphPad) and R version 4.1.3. Paired samples were compared using the Wilcoxon signed-rank test, and unpaired samples were compared using the Mann-Whitney U test. In all dot plots, horizontal bars represent median values, and in all figures, significance is denoted as follows: n.s. (not significant), *P < 0.05, **P < 0.01, ***P < 0.001, ****P < 0.0001.

## Supplementary Material

Supplementary Material

## Data Availability

Expression matrices from single-cell sequencing experiments have been deposited to ArrayExpress under the accession number E-MTAB-12716. The R code used for single-cell analyses are available via Zenodo (https://doi.org/10.5281/zenodo.7594134). All other data needed to support the conclusions of the paper are present in the paper or the Supplementary Materials.

## One Sentence Summary

SARS-CoV-2 vaccination enhances spike-specific T cell functionality after COVID-19.
