# Supplementary material for: SARS-CoV-2 vaccination enhances the effector qualities of spike-specific T cells induced by COVID-19
Source: Sci Immunol. Author manuscript; Available in PMC 2024 Feb 1. (PMC7615587; doi:10.1126/sciimmunol.adh0687)
Supplement: Supplementary Material [file EMS192724-supplement-Supplementary_Materials.pdf]

SUPPLEMENTARY MATERIALS

Fig. S1. Detection of AIM^+ T cells.

Fig. S2. Effect of vaccination on the frequencies of spike-specific T cells.

Fig. S3. Characterization of T cell cytokine production and inhibitory receptor expression.

Fig. S4. Classification of AIM^+ T cell populations sorted for scRNA-seq.

Fig. S5. Protein expression among spike-specific T cells determined via CITE-seq.

Fig. S6. Transcriptomic comparison of spike-specific T cells before and after vaccination.

Fig. S7. Clonal characterization of spike-specific T cells before and after vaccination.

Fig. S8. Transcriptomic signatures of $CD8^+$ T cell clonotypes detected at one or both time points.

Fig. S9. Characterization of hybrid spike-specific T cell responses in donors with CLL.

Fig. S10. Summary of hybrid T cell immunity shaped by infection and vaccination.

Table S1. Summary of donors with a history of mild or severe COVID-19.

Table S2. Summary of donors selected for tetramer analysis.

Table S3. Summary of donors selected for scRNA-seq.

Table S4. Summary of donors with CLL.

Table S5. Summary of healthy vaccinated donors.

Table S6. Surface staining protocol for flow cytometry.

Table S7. Surface and intracellular staining protocol for flow cytometry.

Table S8. Tetramer, surface, and intracellular staining protocol for flow cytometry.

Table S9. Surface and oligo-conjugated antibody staining protocol for single-cell sorting and CITE-seq.

Data file S1. Raw data file for main figures.

Data file S2. Raw data file for supplementary figures.

Data file S3. Lists of differentially expressed genes.

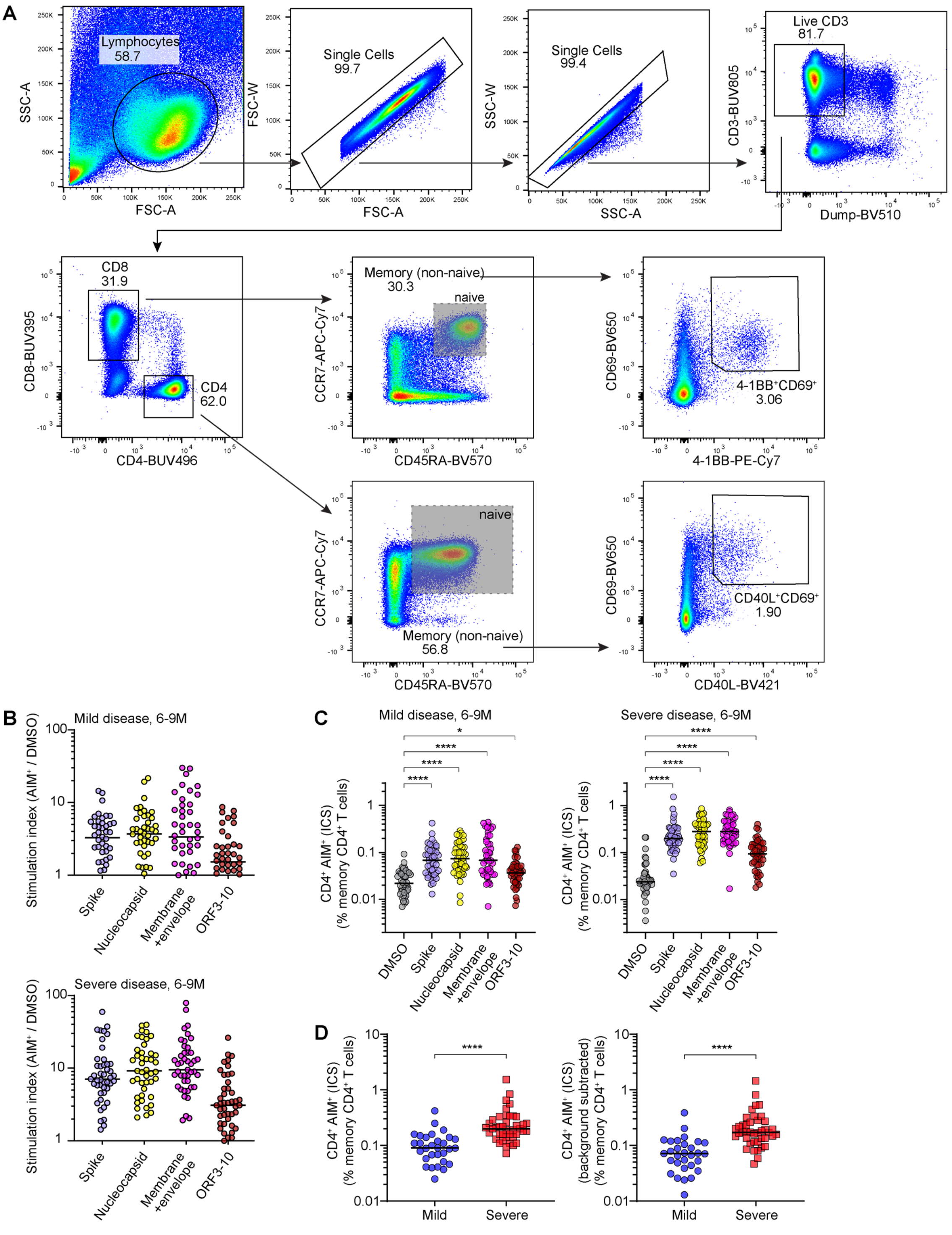

Fig. S1. Detection of AIM⁺ T cells. (**A**) Representative flow cytometric gating strategy showing the identification of AIM⁺ memory CD4⁺ and CD8⁺ T cells via flow cytometry. (**B**) Stimulation indices of individual donor responses at 6–9M. (**C**) Frequencies of intracellular AIM⁺ memory CD4⁺ T cells after DMSO and peptide pool stimulation. (**D**) Frequencies of AIM⁺ memory CD4⁺ T cells measured by intracellular staining, plotted with raw values (left) and DMSO background subtracted values (right). Statistical significance was determined by Mann–Whitney U test (C and D). *$P < 0.05$, **$P < 0.01$, ***$P < 0.001$, ****$P < 0.0001$. Bars shown median.

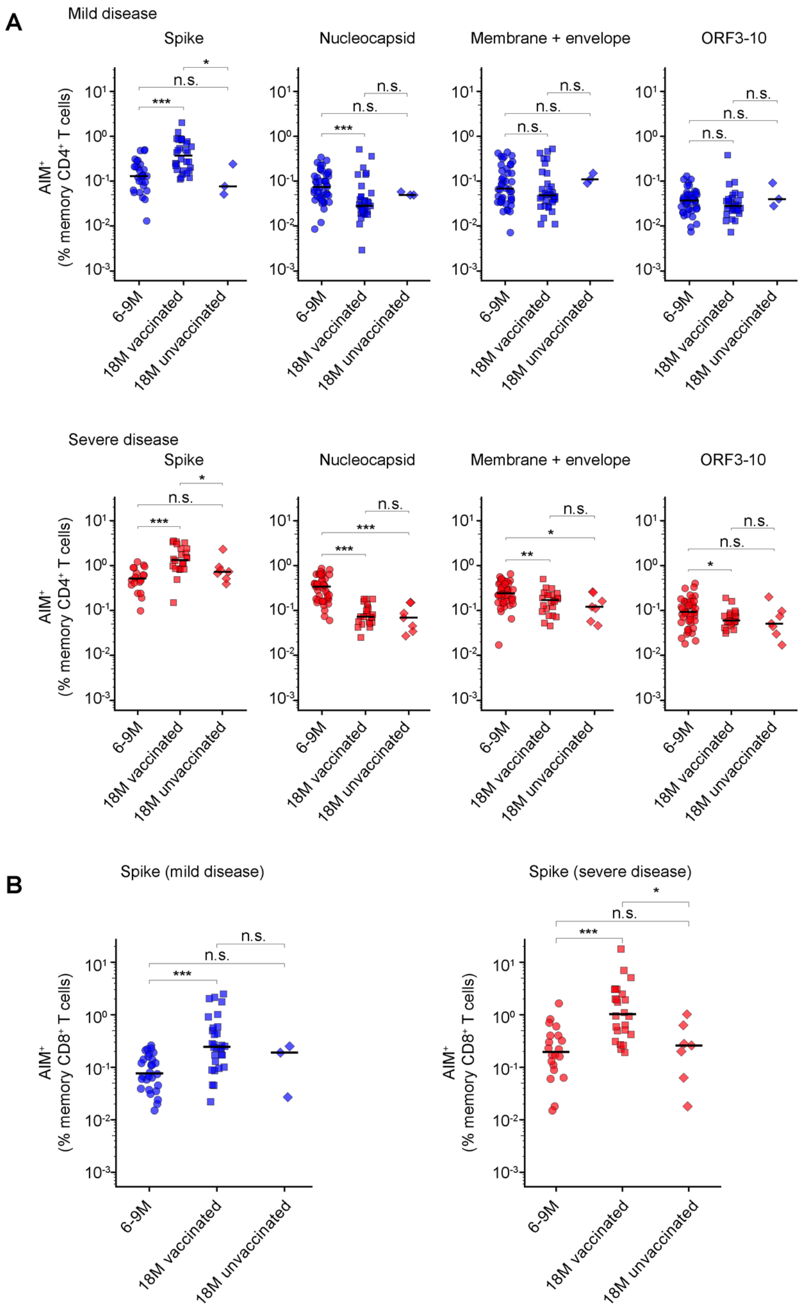

Fig. S2. Effect of vaccination on the frequency of spike-specific T cells. (**A**) Frequencies of AIM$^+$ memory CD4$^+$ T cells targeting different regions of SARS-CoV-2. (**B**) Frequencies of AIM$^+$ memory CD8$^+$ T cells targeting the spike protein of SARS-CoV-2. Statistical significance was determined by Mann–Whitney U test (A and B). n.s. = $P > 0.05$, *$P < 0.05$, **$P < 0.01$, ***$P < 0.001$, ****$P < 0.0001$. Bars shown median.

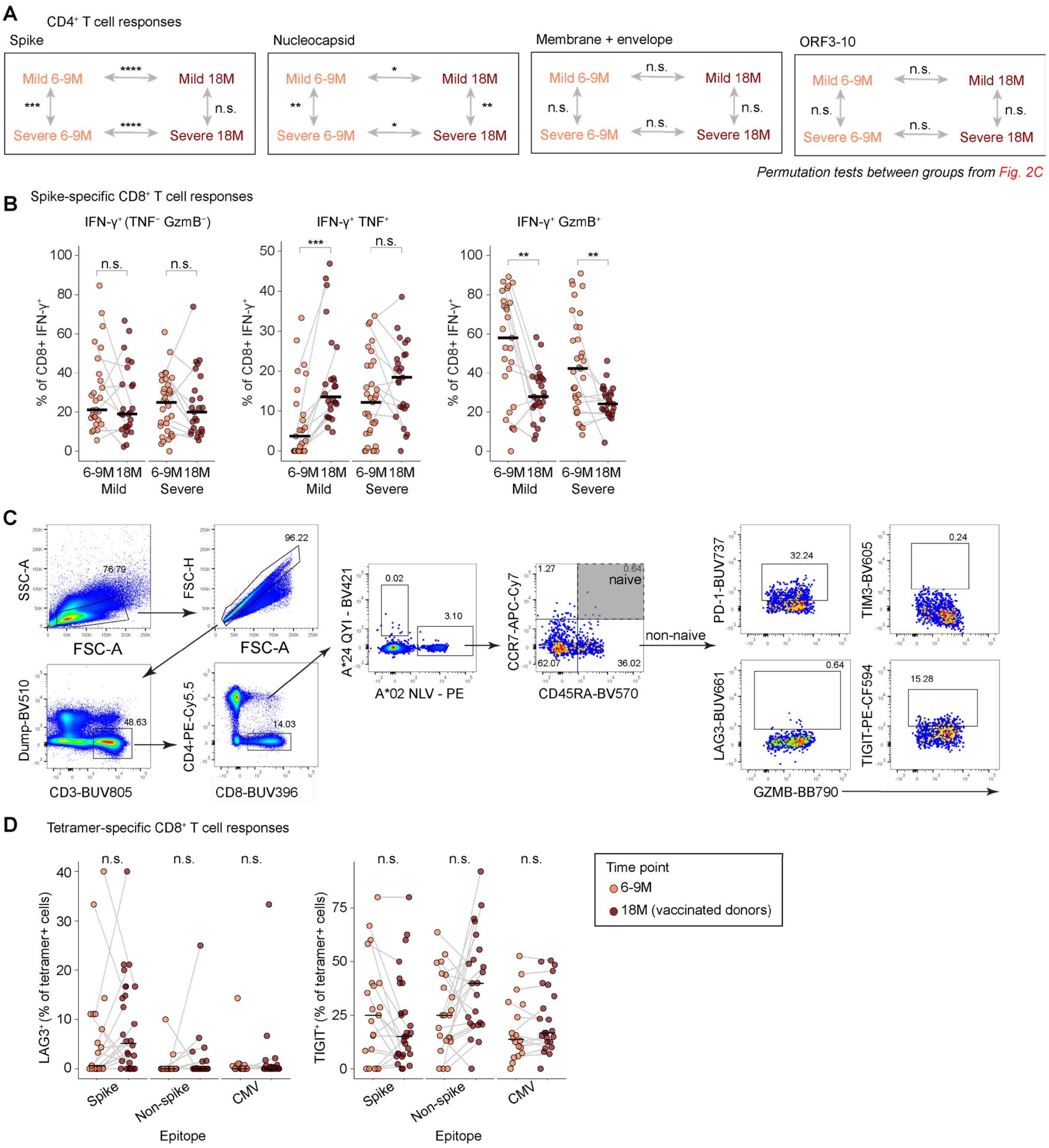

Fig. S3. Characterization of T cell cytokine production and inhibitory receptor expression. (**A**) Permutation test comparisons of cytokine expression profiles among AIM$^+$ memory CD4$^+$ T cells (related to Fig. 2C). (**B**) Percentages of IFN-γ$^+$ spike-specific memory CD8$^+$ T cells with polyfunctional cytokine and cytotoxic molecule expression after peptide stimulation. (**C**) Representative flow cytometry plots showing the identification of inhibitory receptor expression among tetramer-binding CD8$^+$ T cells. (**D**) Percentages of tetramer-binding CD8$^+$ T cells with inhibitory receptor expression. Statistical significance was determined by permutation test (A) and Mann–Whitney U test (B and D). n.s. = $P > 0.05$, *$P < 0.05$, **$P < 0.01$, ***$P < 0.001$, ****$P < 0.0001$. Bars shown median.

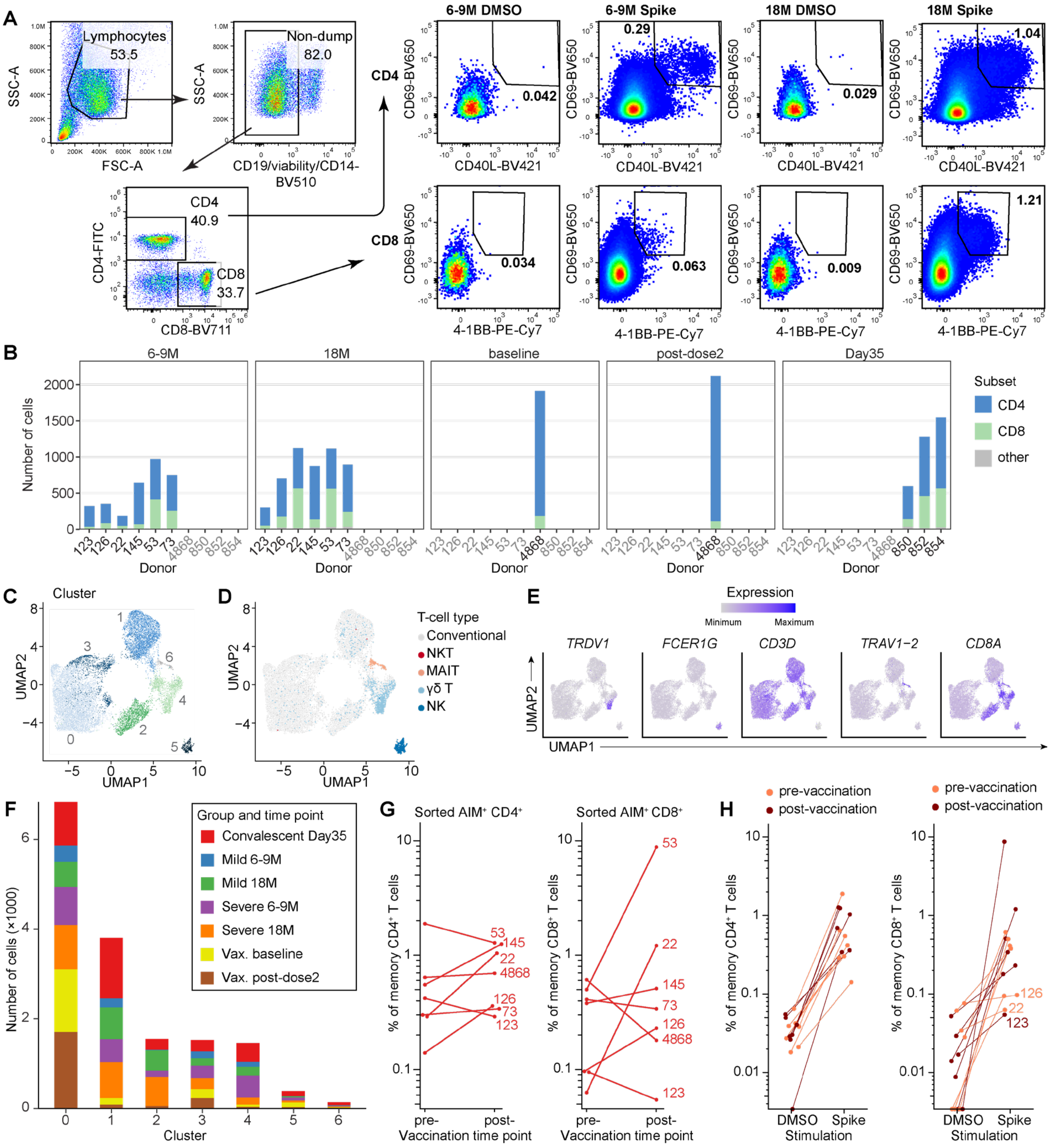

Fig. S4. Classification of AIM⁺ populations sorted for scRNA-seq. (**A**) Representative flow cytometric gating strategy for the identification and sorting of AIM⁺ CD4⁺ and CD8⁺ T cells for scRNA-seq. (**B**) Distribution of conventional AIM⁺ cells with CD4 or CD8 protein expression separated by donor and time point. Cells classified as 'other' either lacked expression of CD4 and CD8 or expressed both CD4 and CD8. (**C**) UMAP and clustering of all sorted cells, including NK cells and unconventional T cells. (**D**) Classification of NK cells and unconventional T cells. (**E**) Expression of transcripts corresponding to conventional and unconventional T cell subsets. (**F**) Distribution of donor groups and time points across each UMAP cluster. (**G**) Frequencies of sorted AIM⁺ memory CD4⁺ and CD8⁺ T cells after spike peptide pool stimulation. (**H**) Comparison of frequencies of AIM⁺ populations after DMSO and spike peptide pool stimulation.

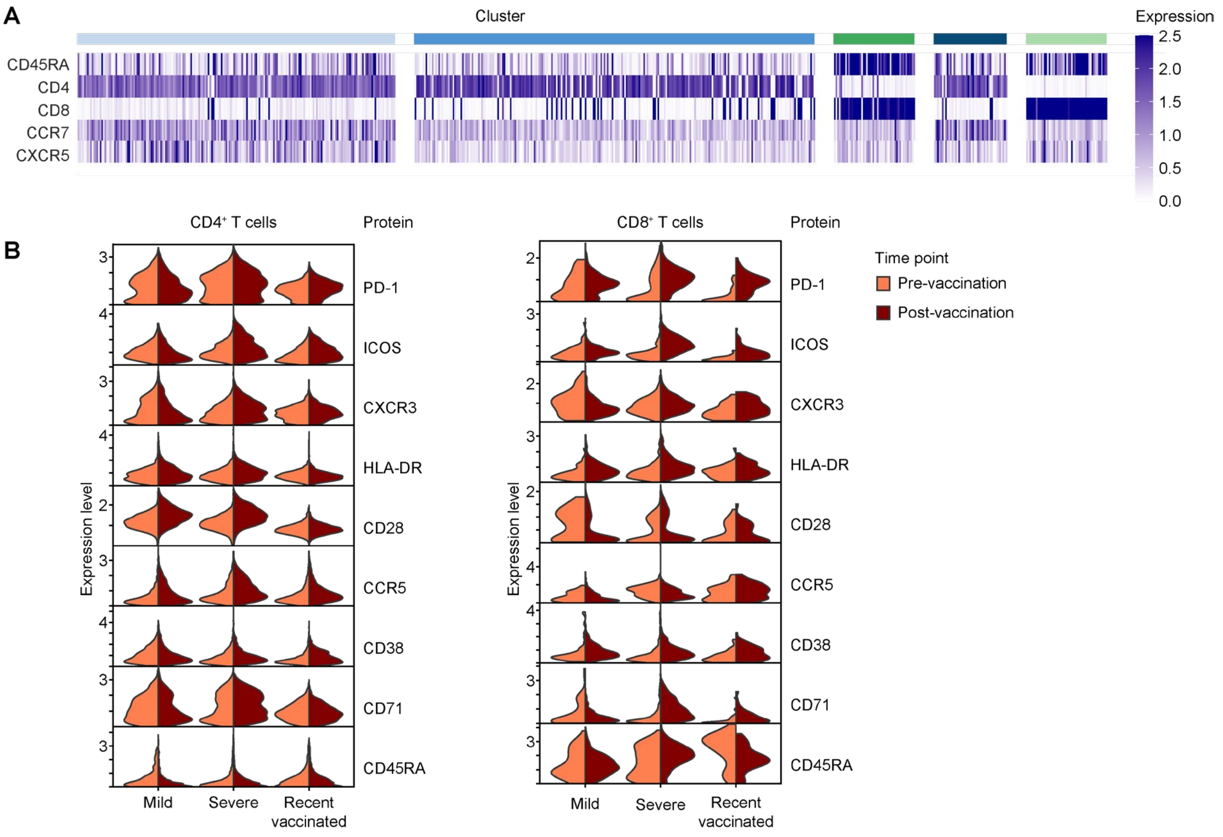

Fig. S5. Protein expression among spike-specific T cells determined via CITE-seq. (**A**) Heatmap showing protein expression measured via CITE-seq using a reduced panel for convalescent donors sampled on day 35. (**B**) Violin plots showing the expression of activation markers separated by donor group and time point.

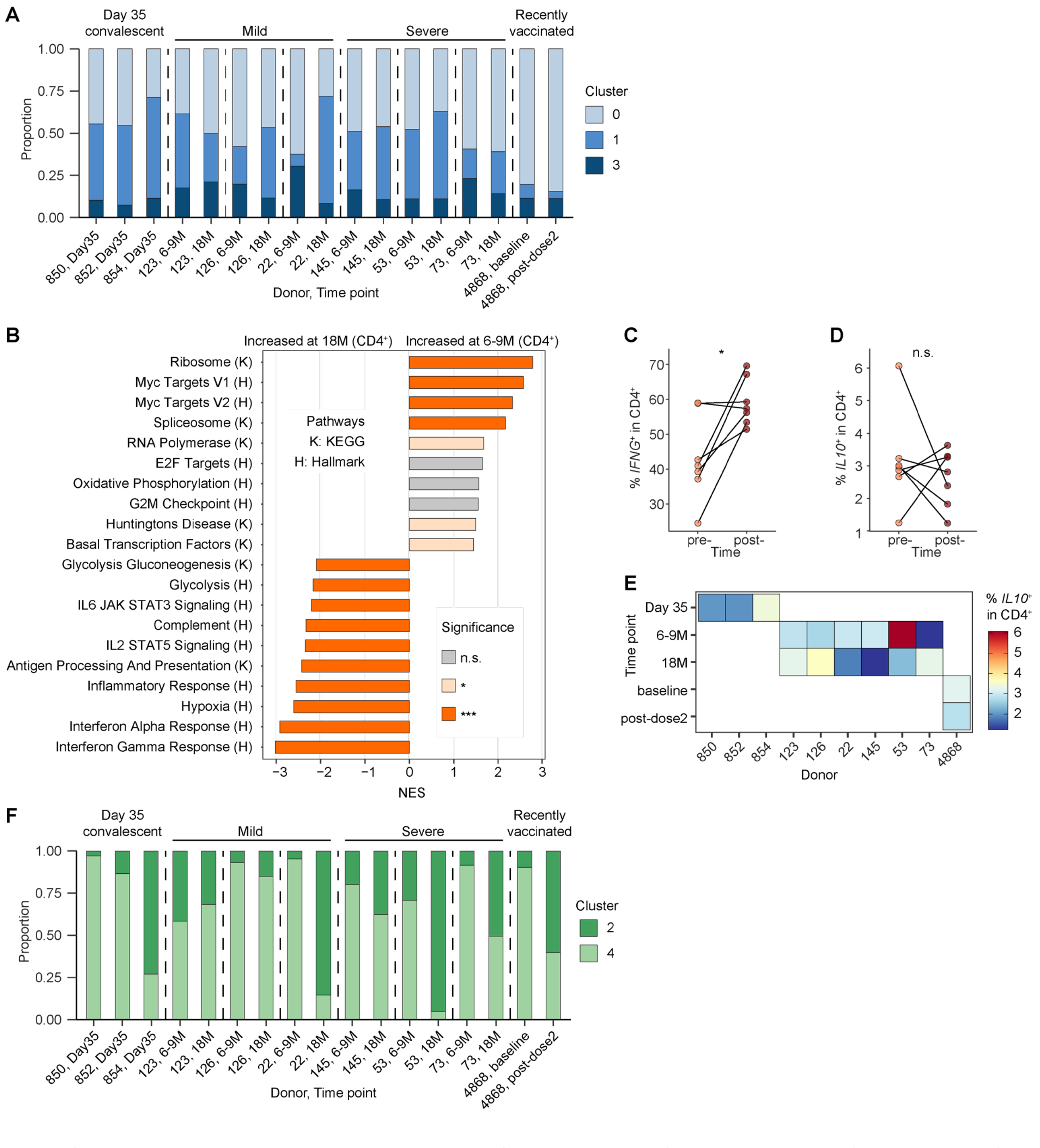

Fig. S6. Transcriptomic comparison of spike-specific T cells before and after vaccination. (**A**) Proportions of AIM⁺ CD4⁺ T cells from individual donors belonging to each CD4⁺ T cell cluster. (**B**) GSEA summary of differentially expressed genes between CD4⁺ T cells at 6-9M versus 18M. (**C**) Dot plots of the percentages of CD4⁺ T cells from mild, severe and recently vaccinated donors with expression of *IFNG*. (**D**) Dot plots of the percentages of CD4⁺ T cells from mild, severe and recently vaccinated donors with expression of *IL10*. (**E**) Percentages of CD4⁺ T cells with expression of *IL10*. (**F**) Proportions of AIM⁺ CD8⁺ T cells from individual donors belonging to each CD8⁺ T cell cluster. Statistical significance was determined by Broad GSEA test (B) and Mann–Whitney U test (C and D). Adjusted p-values calculated using the Benjamini–Hochberg (B) method. n.s. = $P > 0.05$, *$P < 0.05$, **$P < 0.01$, ***$P < 0.001$

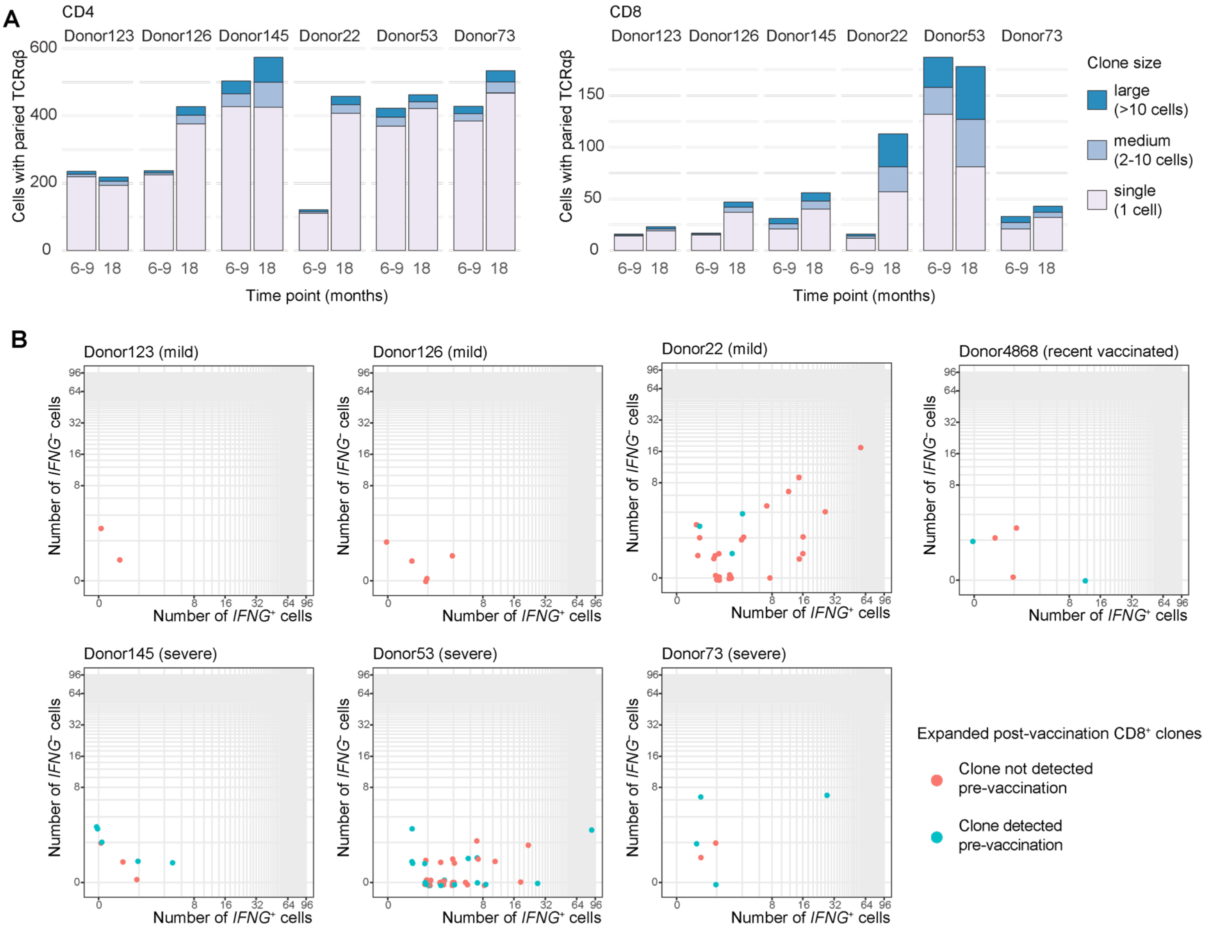

Fig. S7. Clonal characterization of spike-specific T cells before and after vaccination. (**A**) Proportions of $CD4^+$ and $CD8^+$ T cells classified by the degree of clonal expansion. (**B**) *IFNG* expression among expanded $CD8^+$ T cell clonotypes after vaccination separated by donor.

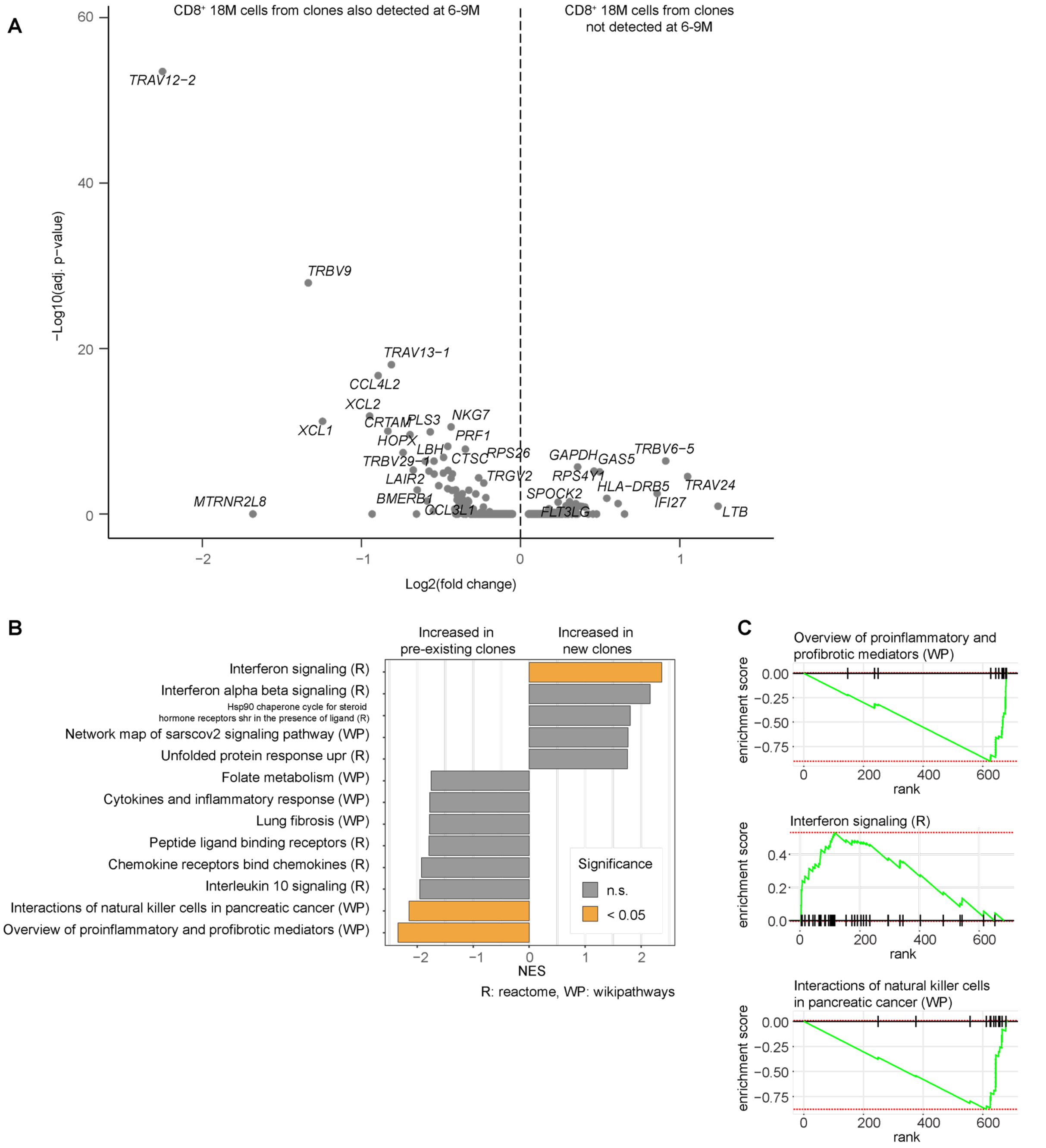

Fig. S8. Transcriptomic signatures of CD8⁺ T cell clonotypes detected at one or both time points. (**A**) Volcano plot showing differentially expressed genes between existing and newly detected CD8⁺ T cell clonotypes at 18M. (**B**) GSEA summary of differentially expressed genes between existing and newly detected CD8⁺ T cell clonotypes at 18M. (**C**) GSEA plot showing significantly enriched pathways at 18M. WP: WikiPathways; R: Reactome. Statistical significance was determined by Mann–Whitney U test (A) and Broad GSEA test (C) and. Adjusted p-values calculated using the Bonferroni method (A). n.s. = $P > 0.05$, $*P < 0.05$

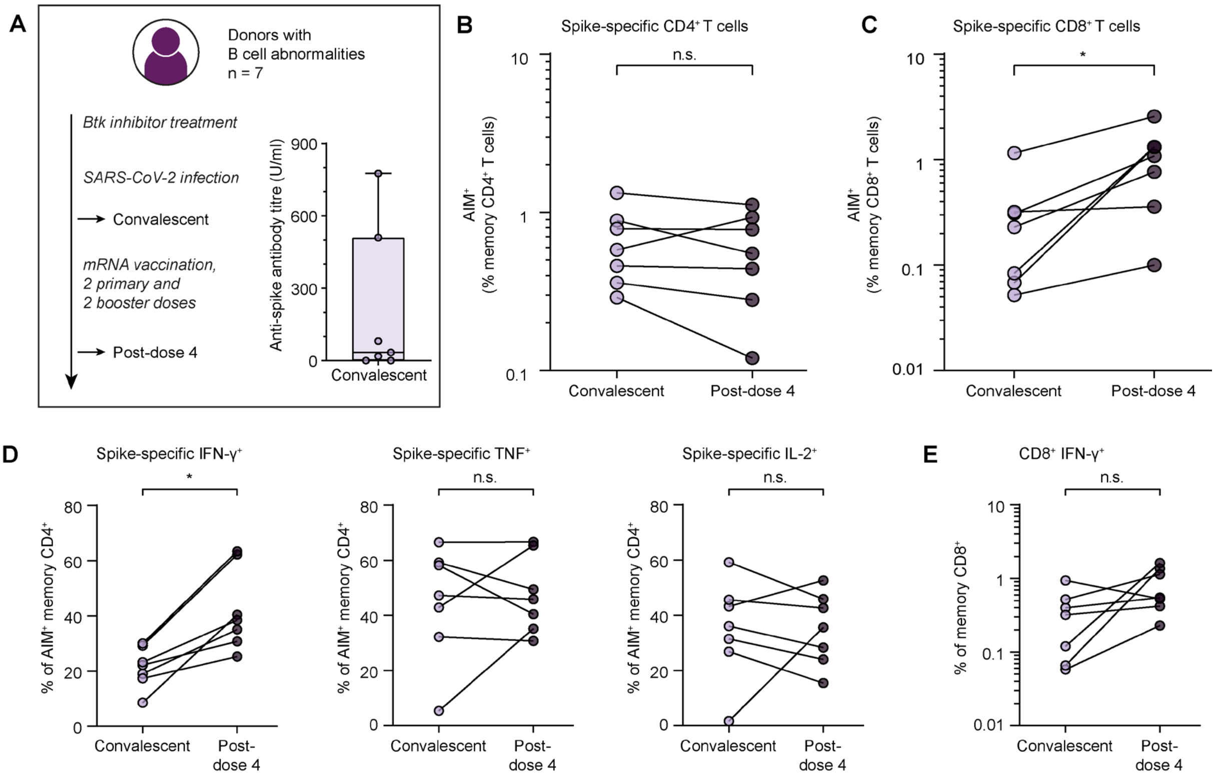

Fig. S9. Characterization of hybrid spike-specific T cell responses in donors with CLL. (**A**) Overview of donors and sampling time points from a cohort of patients undergoing treatment for CLL. (**B**) Frequencies of AIM$^+$ memory CD4$^+$ T cells. (**C**) Frequencies of AIM$^+$ memory CD8$^+$ T cells. (**D**) Percentages of AIM$^+$ memory CD4$^+$ T cells expressing cytokines. (**E**) Percentages of total memory CD8$^+$ T cells expressing IFN-γ. Statistical significance was determined by paired Wilcoxon Signed-Rank Test (B, C, D, E). n.s. = $P > 0.05$, $^*P < 0.05$

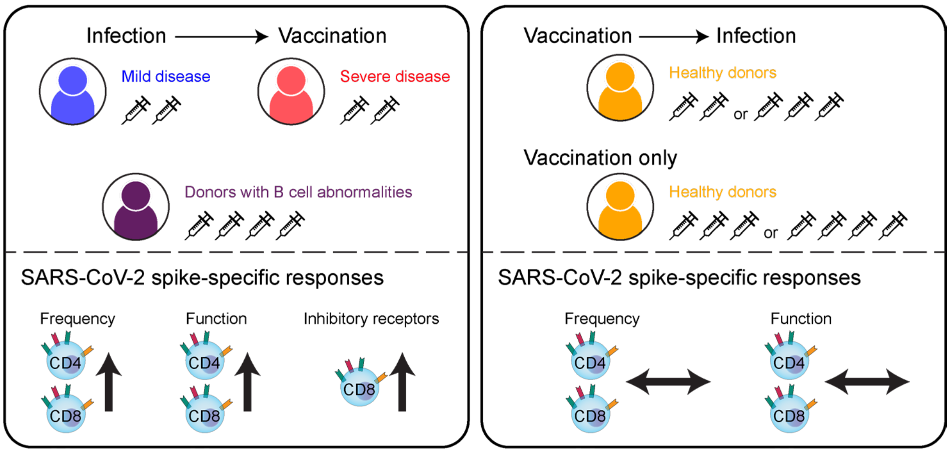

Fig. S10. Summary of hybrid T cell immunity shaped by infection and vaccination. Schematic representation of the key findings.

Table S1. Summary of donors with a history of mild or severe disease.

Donor group and time point	Characteristic	Value
Mild (non-hospitalized)	Total number	50
	Number with paired time points	31
6–9 months convalescence	Total number	44
	Unvaccinated	44
	Median age at infection (range)	54.5 (43−78)
	Male / Female	33 / 11 (75/25 %)
18 months convalescence	Total number	37
	Unvaccinated	3
	Vaccinated	34
	Vaccine platform (Comirnaty, SpikeVax, Vaxzevria, unknown)	27, 3, 2, 2
	Median age at infection (range)	57 (43−78)
	Male / Female	28 / 9 (76/24 %)
Severe (hospitalized)	Total number	53
	Number with paired time points	24
6–9 months convalescence *	Total number	45
	Unvaccinated	45
	Median age at infection (range)	57 (33−68)
	Male / Female *	34 / 7 (76/16 %)
	Admitted to ICU	26
	Required ventilator	21
18 months convalescence	Total number	32
	Unvaccinated	7
	Vaccinated	25
	Vaccine platform (Comirnaty, SpikeVax, Vaxzevria, unknown)	16, 6, 0, 3
	Median age at infection (range)	57.5 (33−76)
	Male / Female	23 / 9 (72/28 %)
	Admitted to ICU	18
	Required ventilator	15

The exact date of infection or vaccination was unavailable for four donors with mild disease and two donors with severe disease. *Clinical information was unavailable from four donors. ICU: intensive care unit.

Table S2. Summary of donors selected for tetramer analysis.

Donor group and time point	Characteristic	Value
Mild (non-hospitalized)<br>• Paired 6–9M and 18M time points for each donor<br>• All donors vaccinated at the 18M time point	Total number	14
	Median age at infection (range)	54 (43−66)
	Male / Female	10 / 4
	HLA combinations	
	A24	3
	A2 A24	6
	A2 B7	2
	A24 B7	2
	A2 A24 B7	1
Severe (hospitalized)<br>• Paired 6–9M and 18M time points for each donor<br>• All donors vaccinated at the 18M time point	Total number	14
	Median age at infection (range)	57.5 (33−68)
	Male / Female	11 / 3
	HLA combinations	
	A2	5
	A24	5
	A2 A24	2
	A2 B7	1
	A2 A24 B7	1

Table S3. Summary of donors selected for single-cell RNA-sequencing (scRNA-seq).

Donor group and time point	Characteristic	Value
Mild<br>• Non-hospitalized<br>• 6–9 months convalescence<br>• 18 months convalescence	Donor IDs	22, 123, 126
	Age	67, 59, 57
	Sex	M, M, M
	Vaccine platform	
	Vaxzevria (Donor IDs)	22
	Comirnaty (Donor IDs)	123, 126
Severe<br>• Non-hospitalized<br>• 6–9 months convalescence<br>• 18 months convalescence	Donor IDs	53, 73, 145
	Age	56, 62, 56
	Sex	M, F, F
	Vaccine platform	
	Comirnaty (Donor IDs)	53, 73
	Unknown (Donor IDs)	145
Recently vaccinated<br>• Non-hospitalized<br>• Baseline: 2 weeks before vaccination or 13 months convalescence<br>• Dose 2+: 2 weeks after second dose vaccination or 15 months convalescence	Donor IDs	4868
	Age	54
	Sex	F
	Vaccine platform	
	SpikeVax (Donor IDs)	4868
Recently convalescent<br>• Non-hospitalized<br>• Day 35 convalescence<br>• Unvaccinated	Donor IDs	850, 852, 854
	Unvaccinated	3
	Age	Unavailable
	Sex	F, F, M

Table S4. Summary of donors with B cell abnormalities (CLL).

Donor group and time point	Characteristic	Value
B cell abnormality<br>• Treated with BTK inhibition for CLL<br>• Convalescent<br>• Post-dose 4	Total number	7
	Median age at convalescent time point (range)	69 (46−77)
	Male / Female	4 / 3
	Hospitalized	6

CLL: chronic lymphocytic leukemia; BTK: Bruton's tyrosine kinase

Table S5. Summary of healthy vaccinated donors.

Donor group and time point	Characteristic	Value
Vaccinated healthy controls<br>• 3 month post-vaccination follow-up (two doses received)<br>• 18 month post-vaccination follow-up (three or four doses received)	Total number	14
With breakthrough infection	Total number	5
	Median age at vaccination (range)	32 (26−55)
	Male / Female	2 / 3
	Vaccine platform	
	Comirnaty only	2
	Comirnaty and SpikeVax	3
	Clinical history	
	Two doses > breakthrough	1
	Two doses > breakthrough > third dose	1
	Three doses > breakthrough	3
Without breakthrough infection<br>• All received four vaccine doses by the 18 month follow-up	Total number	9
	Median age at vaccination (range)	46 (28−59)
	Male / Female	4 / 5
	Vaccine platform	
	Comirnaty only	6
	Comirnaty and SpikeVax	3

Table S6. Surface staining protocol for flow cytometry.

STEP 1: Stain for viability at room temperature for 10 minutes.					
**Marker**	**Fluorophore**	**Supplier**	**Dilution**	**Product number**	**Clone**
LIVE/DEAD Fixable Aqua	For 405 nm excitation	Invitrogen	1X in PBS	L34957	-
STEP 2: Stain for chemokine receptors at 37°C for 10 minutes.					
**Marker**	**Fluorophore**	**Supplier**	**Dilution**	**Product number**	**Clone**
CCR7	APC-Cy7	BioLegend	1:50	353212	G043H7
CCR4	BB700	BD	1:50	566475	1G1
CCR6	BUV737	BD	1:75	612780	11A9
CXCR3	AF647	BioLegend	1:100	353712	G025H7
STEP 3: Stain with remaining antibodies at room temperature for 30 minutes in BD Brilliant Stain Buffer Plus.					
**Marker**	**Fluorophore**	**Supplier**	**Dilution**	**Product number**	**Clone**
CD40L	BV421	BioLegend	1:25	310824	24-31
4-1BB	PE-Cy7	BioLegend	1:25	309818	4B4-1
CD4	BUV496	BD	1:25	612936	SK3
CD14	BV510	BioLegend	1:100	301842	M5E2
CD19	BV510	BioLegend	1:100	302242	HIB19
CD45RA	BV570	BioLegend	1:200	304132	HI100
CD69	BV650	BioLegend	1:50	310934	FN50
CD3	BUV805	BD	1:50	612895	UCHT1
CD8	BUV395	BD	1:250	563795	RPA-T8
STEP 4: Wash and fix cells in 1% paraformaldehyde.					

Table S7. Surface and intracellular staining protocol for flow cytometry.

STEP 1: Stain for chemokine receptors at 37°C for 10 minutes.					
**Marker**	**Fluorophore**	**Supplier**	**Dilution**	**Product number**	**Clone**
CCR7	APC-Cy7	BioLegend	1:50	353212	G043H7
CCR4	BB700	BD	1:50	566475	1G1
CCR6	BUV737	BD	1:75	612780	11A9
CXCR3	AF647	BioLegend	1:50	353712	G025H7
STEP 2: Stain with surface antibodies and viability dye at room temperature for 30 minutes in BD Brilliant Stain Buffer Plus.					
**Marker**	**Fluorophore**	**Supplier**	**Dilution**	**Product number**	**Clone**
PD-1	BV711	BioLegend	1:25	329928	EH12.2H7
CD4	BUV496	BD	1:25	612936	SK3
CD14	BV510	BioLegend	1:100	301842	M5E2
CD19	BV510	BioLegend	1:100	302242	HIB19
CD45RA	BV570	BioLegend	1:200	304132	HI100
CD8	BUV395	BD	1:250	563795	RPA-T8
LIVE/DEAD Fixable Aqua	For 405 nm excitation	Invitrogen	1:1667	L34957	-
CD38	APC-R700	BD	1:50	564979	HIT2
STEP 3: Fix and permeabilize with FoxP3 Transcription Factor Staining Buffer Set (Invitrogen, #00-5523-00) according to the provided protocol.					
STEP 4: Stain with intracellular antibodies at room temperature for 30 minutes in BD Brilliant Stain Buffer Plus and 1X Permeabilization Buffer.					
**Marker**	**Fluorophore**	**Supplier**	**Dilution**	**Product number**	**Clone**
CD40L	BV421	BioLegend	1:25	310824	24-31
IL-17A	eFluor660	Invitrogen	1:25	50-7178-42	eBio64CAP17
IL-2	PE-Dazzle594	BioLegend	1:33	500344	MQ1-17H12
4-1BB	PE-Cy7	BioLegend	1:100	309818	4B4-1
TNFa	BV650	BD	1:166	563418	MAb11
CD3	BUV805	BD	1:250	612895	UCHT1
CD69	BUV563	BD	1:200	748764	FN50
IFN-γ	PE	BioLegend	1:400	506507	B27
Granzyme B	BB790	BD	1:500	624296	GB11
STEP 5: Wash and fix cells in 1% paraformaldehyde.					

Table S8. Tetramer, surface and intracellular staining protocol for flow cytometry.

<table>
<tr><td colspan="6">STEP 1: Incubate with dasatinib at room temperature for 10 minutes.</td></tr>
<tr><th>Reagent</th><th>Fluorophore</th><th>Supplier</th><th>Final conc.</th><th>Product number</th><th>Clone</th></tr>
<tr><td>Dasatinib</td><td>-</td><td>STEMCELL</td><td>50 µM</td><td>73082</td><td>-</td></tr>
<tr><td colspan="6">STEP 2: Incubate with one relevant PE tetramer/tetramer pool at room temperature for 20 minutes. Each tetramer should be equivalent to 0.2 µl of pMHC (0.5 µg/ml, total 10µl volume).</td></tr>
<tr><th colspan="4">Reagent</th><th colspan="2">Fluorophore</th></tr>
<tr><td colspan="4">Tetramer pool consisting of:
SARS-CoV-2 nucleocapsid A*0201 LLLDRLNQL
SARS-CoV-2 ORF3a A*0201 ALSKGVHFV
SARS-CoV-2 ORF3 A*0201 LLYDANYFL</td><td colspan="2">PE</td></tr>
<tr><td colspan="4">SARS-CoV-2 nucleocapsid B*0702 SPRWYFYYL</td><td colspan="2">PE</td></tr>
<tr><td colspan="4">CMV pp65 B*0702 TPRVTGGGAM</td><td colspan="2">PE</td></tr>
<tr><td colspan="4">CMV pp65 A*0201 NLVPMVATV</td><td colspan="2">PE</td></tr>
<tr><td colspan="6">STEP 3: Incubate with one relevant BV421 tetramer at room temperature for 20 minutes.</td></tr>
<tr><th colspan="4">Reagent</th><th colspan="2">Fluorophore</th></tr>
<tr><td colspan="4">SARS-CoV-2 spike A*0201 YLQPRTFLL</td><td colspan="2">BV421</td></tr>
<tr><td colspan="4">SARS-CoV-2 spike A*2402 QYIKWPWYI</td><td colspan="2">BV421</td></tr>
<tr><td colspan="4">SARS-CoV-2 spike B*0702 SPRRARSVA</td><td colspan="2">BV421</td></tr>
<tr><td colspan="4">CMV pp65 A*0201 NLVPMVATV</td><td colspan="2">BV421</td></tr>
<tr><td colspan="6">STEP 4: Wash cells. Stain for viability at room temperature for 10 minutes.</td></tr>
<tr><th>Marker</th><th>Fluorophore</th><th>Supplier</th><th>Dilution</th><th>Product number</th><th>Clone</th></tr>
<tr><td>LIVE/DEAD Fixable Aqua</td><td>For 405 nm excitation</td><td>Invitrogen</td><td>1X in PBS</td><td>L34957</td><td>-</td></tr>
<tr><td colspan="6">STEP 5: Stain for chemokine receptors at 37°C for 10 minutes.</td></tr>
<tr><th>Marker</th><th>Fluorophore</th><th>Supplier</th><th>Dilution</th><th>Product number</th><th>Clone</th></tr>
<tr><td>CCR7</td><td>APC-Cy7</td><td>BioLegend</td><td>1:50</td><td>353212</td><td>G043H7</td></tr>
<tr><td>CXCR3</td><td>PE-Cy5</td><td>BioLegend</td><td>1:200</td><td>353756</td><td>G025H7</td></tr>
<tr><td>CX3CR1</td><td>BUV615</td><td>BioLegend</td><td>1:100</td><td>750690</td><td>2A9-1</td></tr>
<tr><td colspan="6">STEP 6: Stain with surface antibodies at room temperature for 30 minutes in BD Brilliant Stain Buffer Plus.</td></tr>
<tr><th>Marker</th><th>Fluorophore</th><th>Supplier</th><th>Dilution</th><th>Product number</th><th>Clone</th></tr>
<tr><td>CD8</td><td>BUV396</td><td>BioLegend</td><td>1:200</td><td>563795</td><td>RPA-T8</td></tr>
<tr><td>CD38</td><td>BUV496</td><td>BD</td><td>1:200</td><td>612946</td><td>HIT2</td></tr>
<tr><td>LAG3</td><td>BUV661</td><td>BD</td><td>1:200</td><td>624285</td><td>T47-530</td></tr>
<tr><td>PD-1</td><td>BUV737</td><td>BD</td><td>1:50</td><td>612791</td><td>EH12.1</td></tr>
<tr><td>CD3</td><td>BUV805</td><td>BD</td><td>1:50</td><td>612895</td><td>UCHT1</td></tr>
<tr><td>CD14</td><td>BV510</td><td>BioLegend</td><td>1:100</td><td>301842</td><td>M5E2</td></tr>
<tr><td>CD19</td><td>BV510</td><td>BioLegend</td><td>1:100</td><td>302242</td><td>HIB19</td></tr>
<tr><td>CD45RA</td><td>BV570</td><td>BioLegend</td><td>1:200</td><td>304132</td><td>HI100</td></tr>
<tr><td>TIM3</td><td>BV605</td><td>BioLegend</td><td>1:100</td><td>502936</td><td>344823</td></tr>
<tr><td>HLA-DR</td><td>BV650</td><td>BD</td><td>1:100</td><td>564231</td><td>G46-6</td></tr>
<tr><td>TIGIT</td><td>PE-Dazzle594</td><td>BioLegend</td><td>1:100</td><td>372715</td><td>A15153G</td></tr>
<tr><td>CD127</td><td>BB630</td><td>BD</td><td>1:100</td><td>Custom</td><td>HIL-7R-M21</td></tr>
<tr><td>CD27</td><td>BV786</td><td>BioLegend</td><td>1:50</td><td>302832</td><td>O323</td></tr>
<tr><td>CD4</td><td>PE-Cy5.5</td><td>Invitrogen</td><td>1:400</td><td>35-0042-82</td><td>RMA-4.5</td></tr>
<tr><td>CD95</td><td>BB700</td><td>BioLegend</td><td>1:50</td><td>305634</td><td>DX2</td></tr>
<tr><td>CD39</td><td>BV711</td><td>BioLegend</td><td>1:100</td><td>328228</td><td>A1</td></tr>
<tr><td colspan="6">STEP 5: Wash and fix cells in 1% paraformaldehyde.</td></tr>
</table>

Table S9. Surface and oligo-conjugated antibody staining protocol for single-cell sorting and CITE-seq.

STEP 1: Stain for viability at room temperature for 10 minutes.					
**Marker**	**Fluorophore**	**Supplier**	**Dilution**	**Product number**	**Clone**
LIVE/DEAD Fixable Aqua	For 405 nm excitation	Invitrogen	1X in PBS	L34957	-
STEP 2: Stain for chemokine receptors at 37°C for 10 minutes.					
**Marker**	**Conjugate**	**Supplier**	**Dilution**	**Product number**	**Clone**
CCR7	TotalSeq-C0148	BioLegend	1:300	353251	G043H7
CXCR5	TotalSeq-C0144	BioLegend	1:500	356939	J252D4
CXCR3	TotalSeq-C0140	BioLegend	1:500	353747	G025H7
CX3CR1	TotalSeq-C0179	BioLegend	1:500	355705	K0124E1
CCR4	TotalSeq-C0071	BioLegend	1:500	359425	L291H4
CCR5	TotalSeq-C0141	BioLegend	1:500	359137	J418F1
CCR6	TotalSeq-C0143	BioLegend	1:500	353440	G034E3
CXCR6	TotalSeq-C0804	BioLegend	1:500	356023	K041E5
STEP 3: Stain with remaining antibodies at room temperature for 30 minutes in BD Brilliant Stain Buffer Plus. Use one hashing antibody per sample.					
**Marker**	**Fluorophore**	**Supplier**	**Dilution**	**Product number**	**Clone**
CD40L	BV421	BioLegend	1:25	310824	24-31
4-1BB	PE-Cy7	BioLegend	1:25	309818	4B4-1
CD4	FITC	BD	1:25	345768	SK3
CD14	BV510	BioLegend	1:100	301842	M5E2
CD19	BV510	BioLegend	1:100	302242	HIB19
CD69	BV650	BioLegend	1:50	310934	FN50
CD8	BV711	BioLegend	1:50	301044	RPA-T8
**Marker**	**Conjugate**	**Supplier**	**Dilution**	**Product number**	**Clone**
CD4	TotalSeq-C0072	BioLegend	1:1250	300567	RPA-T4
CD8	TotalSeq-C0046	BioLegend	1:10000	344753	SK1
CD45RA	TotalSeq-C0063	BioLegend	1:4000	304163	HI100
CD127	TotalSeq-C0390	BioLegend	1:333	351356	A019D5
CD27	TotalSeq-C0154	BioLegend	1:500	302853	O323
PD-1	TotalSeq-C0088	BioLegend	1:500	329963	EH12.2H7
ICOS	TotalSeq-C0171	BioLegend	1:500	313553	C398.4A
HLA-DR	TotalSeq-C0159	BioLegend	1:500	307663	L243
CD122	TotalSeq-C0246	BioLegend	1:500	339021	TU27
CD28	TotalSeq-C0386	BioLegend	1:500	302963	CD28.2
CD95	TotalSeq-C0156	BioLegend	1:500	305651	DX2
CD38	TotalSeq-C0389	BioLegend	1:500	303543	HIT2
CD71	TotalSeq-C0394	BioLegend	1:500	334125	CY1G4
**Hashtag**	**Conjugate**	**Supplier**	**Dilution**	**Product number**	**Clone**
Hashtag 1	TotalSeq-C0251	BioLegend	1:100	394661	LNH-94; 2M2
Hashtag 2	TotalSeq-C0252	BioLegend	1:100	394663	LNH-94; 2M2
Hashtag 3	TotalSeq-C0253	BioLegend	1:100	394665	LNH-94; 2M2
Hashtag 4	TotalSeq-C0254	BioLegend	1:100	394667	LNH-94; 2M2
